# Distinct contributions of foveal and extrafoveal visual information to emotion judgments and gaze behavior for faces

**DOI:** 10.1167/jov.25.8.4

**Published:** 2025-07-02

**Authors:** Anthony P. Atkinson, Nazire Duran, Abigail Skraga, Anita Winterbottom, Jack D. Wright

**Affiliations:** 1 Department of Psychology, Durham University, Durham, UK

**Keywords:** facial expression, emotion recognition, peripheral vision, eye movements, gaze decoding, recurrence quantification analysis

## Abstract

The precise contributions of foveal and extrafoveal visual processing to facial emotion recognition and to how individuals gaze at faces remain poorly understood. We used gaze-contingent masking and windowing to control foveal and extrafoveal inputs while observers (*N* = 35) classified the emotion (anger, disgust, fear, surprise, sadness) on face images. Emotion classification performance was substantially reduced by the absence of extrafoveal information but was unaffected by the absence of foveal information. Gaze decoding showed that fixation patterns discriminated viewed emotion categories regardless of whether either foveal or extrafoveal information was absent or both were present, more so when observers provided correct responses. Although fixations clustered around the eyes, nose, and upper mouth, emotion-specific biases in fixation densities aligned with regions previously identified as emotion diagnostic, and, for trials with incorrect responses, with locations informative of the most confused emotion. Even without extrafoveal information, necessitating top–down guidance of gaze, fixations were biased to these same emotion-informative regions. Yet, the spatiotemporal sequencing of fixations differed in the absence versus presence of extrafoveal information. Fixation patterns also predicted stimulus presentation conditions, most evident in differences due to the absence versus presence of extrafoveal rather than foveal inputs. Thus, where one looks on a face impacts the ability to determine its emotional expression, not only via the higher resolving power of foveal vision but also by the extrafoveal extraction of task-relevant information and guidance of gaze, and possibly also via the interplay between foveal and extrafoveal vision that underpins presaccadic attention.

## Introduction

Human beings are inherently social creatures, endowed with the remarkable ability to decode and interpret complex social cues, particularly those emanating from the faces of others. Within normal interpersonal distances of approximately 0.45 to 1.2 meters (Hall, 1966) another's face will occupy an area of the visual field large enough that not all features can fall simultaneously in the fovea (e.g., Atkinson & Smithson, 2013; Atkinson & Smithson, 2020), a small region of the retina that is preferentially specialized for processing fine spatial detail (Provis, Dubis, Maddess, & Carroll, 2013; Tuten & Harmening, 2021; Wandell, 1995). At a viewing distance of 70 cm, for example, the fovea will capture an area of the face approximately corresponding to the size of the visible part of an eye and eyelids.^1^ We move our eyes around another's face (and visual scenes more generally) to enable high-fidelity vision of specific parts of that face (or scene). This is important because certain facial features are relatively more informative of different social cues; for example, the shape and movement of the mouth can help us interpret what another is saying (e.g., McGurk & MacDonald, 1976; Peelle & Sommers, 2015), the direction of another's eye gaze can signal the state and object of their attention and their intentions (e.g., Argyle & Cook, 1976; Emery, 2000; Langton, Watt, & Bruce, 2000), and the eyes, brow, nose, and mouth regions can differentially signal specific emotions (e.g., Smith, Cottrell, Gosselin, & Schyns, 2005; Smith & Merlusca, 2014; Smith & Schyns, 2009). Not fixating some facial features could result in missing out on important details, not only about those individual features, but possibly also about combinations of or relations between features (i.e., configural and holistic information), which are also important in face processing (e.g., Calder, Young, Keane, & Dean, 2000; Hayward, Crookes, Chu, Favelle, & Rhodes, 2016; Richler & Gauthier, 2014; Tanaka, Kaiser, Butler, & Le Grand, 2012; Taubert, Apthorp, Aagten-Murphy, & Alais, 2011; Todorov, Loehr, & Oosterhof, 2010). This is because of the quantitative and qualitative differences between foveal and extrafoveal visual processing—most notably, the varying spatial resolution of visual processing across the retina and the effects of visual crowding^2^ in extrafoveal vision (e.g., Rosenholtz, 2016; Stewart, Valsecchi, & Schütz, 2020; Strasburger, Rentschler, & Jüttner, 2011).

In this study, we address three questions: (a) What are the distinct contributions of foveal and extrafoveal visual processing to the ability to decipher what emotion a face is expressing? (b) When deciding what emotion a face is expressing, do people direct their gaze to the most informative facial features for the emotion or emotions in question? (c) What are the distinct contributions of foveal and extrafoveal vision to determining where people direct their gaze on faces (during emotion judgments)? In the following paragraphs, we discuss each of these related questions in turn.

### What are the distinct contributions of foveal and extrafoveal visual processing to the ability to decipher what emotion a face is expressing?

In previous research (Atkinson & Smithson, 2020; Duran & Atkinson, 2021), we hypothesized that a single fixation on an emotion-distinguishing facial feature would enhance emotion identification performance compared with a single fixation on another part of the face, at least when the viewed face subtends visual angles equivalent to a real face at a normal interpersonal distance and for features for which medium to high spatial frequency information is most informative. Our reasoning was that the differences between foveal and extrafoveal visual processing would lead to differences in emotion classification performance depending on whether the emotion-distinguishing feature appears in foveal or extrafoveal vision. We tested this hypothesis using an enforced brief fixation paradigm, in which faces were presented for a brief time, insufficient for a saccade, at a position that guaranteed that a specific feature (e.g., an eye) fell at the fovea. We found that (a) enforced fixation of the central brow significantly improved emotion classification of angry faces compared with fixation of a cheek or eye (Atkinson & Smithson, 2020; Duran & Atkinson, 2021), although not in all cases (Duran & Atkinson, 2021); (b) enforced fixation of the mouth center significantly improved classification of disgusted and surprised faces compared with fixation of an eye, cheek, or central brow (Duran & Atkinson, 2021); and (c) enforced fixation of the mouth center allowed observers to more accurately distinguish between fearful and surprised expressions (Atkinson & Smithson, 2020; Duran & Atkinson, 2021). These findings are consistent with research showing that mid- to high spatial frequency information at the central brow (and eyebrows) is informative or diagnostic of anger (Smith et al., 2005; Smith & Merlusca, 2014; Smith & Schyns, 2009), that mid- to high spatial frequency information at the mouth (as well as around the lower nose) provides diagnostic information for disgust (Smith et al., 2005; Smith & Merlusca, 2014; Smith & Schyns, 2009), and that the mouth is a diagnostic region for surprise (Smith et al., 2005; Smith & Schyns, 2009) and aids discrimination between fear and surprise particularly (Du, Tao, & Martinez, 2014; Roy-Charland, Perron, Beaudry, & Eady, 2014). Nonetheless, performance in classifying fear and happiness was not influenced by whether the most informative features (eyes and mouth, respectively) were projected foveally or extrafoveally (Atkinson & Smithson, 2020).

The enforced brief fixation paradigm allows one to investigate the distinct contributions of foveal and extrafoveal vision to task performance via the careful control of the loci of fixations on the face image. That control is lost if we present the faces for longer, allowing observers to freely explore the faces and thus make multiple fixations, as they would typically do when viewing faces in images or in the flesh (e.g., Althoff & Cohen, 1999; Bindemann, Scheepers, & Burton, 2009; Blais, Jack, Scheepers, Fiset, & Caldara, 2008; Hsiao & Cottrell, 2008; Mehoudar, Arizpe, Baker, & Yovel, 2014; Varela, Towler, Kemp, & White, 2023; Walker-Smith, Gale, & Findlay, 1977; Yarbus, 1967). Under such more typical viewing conditions, the experimenter must use some other way of controlling what information gets presented inside and outside the fovea. One option, which we chose in the present study, is to gaze-contingently mask either the foveated or the non-foveated region of the image. We had participants classify the emotions displayed in photographic images of faces under four different viewing conditions: an unrestricted viewing condition, in which the whole face was visible for 3 seconds, and 3 gaze-contingent conditions with the same image duration, which were designed to restrict the emotionally expressive parts of the face to either foveal or extrafoveal visual processing. A gaze-contingent blindspot condition allowed only extrafoveal processing of the facial expression by dynamically occluding the foveated parts of the face with an opaque disc. A similar gaze-contingent condition allowed only extrafoveal processing of the facial expression by dynamically occluding the foveated parts of the face with the corresponding part of the neutral expression face for the same individual, rather than with the opaque disc. This neutral expression blindspot condition was introduced to reduce the disruption of configural and holistic processing of the faces caused by the opaque disc occluder in the standard blindspot condition. Finally, a gaze-contingent spotlight condition (e.g., Caldara, Zhou, & Miellet, 2010; Kennedy & Adolphs, 2010; Urtado, Rodrigues, & Fukusima, 2024) allowed only foveal processing of the facial expression by dynamically occluding all but the foveated region of the image.

To the extent that emotion classification performance over multiple fixations depends on foveal visual processing, we expected reduced performance in the blindspot conditions compared with the unrestricted viewing condition. To the extent that emotion classification performance over multiple fixations depends on extrafoveal information, we expected reduced performance in the spotlight condition compared with the unrestricted viewing condition. Yet, masking all but the foveated region eliminates a much larger amount of facial information compared with masking the foveated region and eliminates, rather than slightly reduces, the contemporaneous availability of configural or holistic information, making a direct comparison between these two masked conditions unfair. Thus, although we expected a large decrement in emotion classification performance when the extrafoveal parts of the image were masked, we did not take this as an indication of a critical role for extrafoveal visual processing in emotion recognition comparable to the impact of the blindspot condition upon foveal visual processing. Rather, our primary interest in the spotlight condition was its impact on gaze behavior to the faces (as discussed below).

### When deciding what emotion a face is expressing, do people direct their gaze to the most informative facial features or regions for the emotion or emotions in question?

Gaze behavior to faces in general, under unrestricted viewing conditions, is typically characterized by a first fixation to a location near face center, just below the eyes and slightly to the left of the face midline (Hsiao & Cottrell, 2008; Liu, Zhan, & Wang, 2024; Peterson & Eckstein, 2012). Second and subsequent fixations tend to be more dispersed, encompassing not only the nose/face center but also the eyes and mouth (Bindemann et al., 2009; Hsiao & Cottrell, 2008; Liu et al., 2024; Paparelli et al., 2024). From the third fixation on, a classic triangular or T-shaped pattern of fixations on the eyes, nose, and mouth becomes evident (e.g., Althoff & Cohen, 1999; Bindemann et al., 2009; Blais et al., 2008; Hsiao & Cottrell, 2008; Mehoudar et al., 2014; Peterson, Lin, Zaun, & Kanwisher, 2016; Yarbus, 1967). Although such gaze behavior to faces is typically evident in group average data, it is not always evident in individual observer data: People have idiosyncratic eye movements toward faces that are stable across time and task (Arizpe, Walsh, Yovel, & Baker, 2017; Kanan, Bseiso, Ray, Hsiao, & Cottrell, 2015; Mehoudar et al., 2014; Peterson & Eckstein, 2013; Peterson et al., 2016); cultural differences have also been reported (Blais, Fiset, Roy, Saumure Régimbald, & Gosselin, 2017; Caldara et al., 2010; Fu, Hu, Wang, Quinn, & Lee, 2012; Jack, Blais, Scheepers, Schyns, & Caldara, 2009; Kelly et al., 2011; Miellet, Vizioli, He, Zhou, & Caldara, 2013; Senju, Vernetti, Kikuchi, Akechi, & Hasegawa, 2013).

How, then, does the viewed emotional expression impact eye-gaze behavior toward those faces? More particularly, do people preferentially fixate emotion-informative facial features? Do they do so more when their judgments of the emotion are correct than when they are incorrect? Are incorrect emotion judgments associated with fixations on features that are informative of the selected (incorrect) emotion, rather than of the correct emotion? Existing evidence indicates that fixations cluster on key emotion-informative facial features when observers view images of facial expressions. Which emotion-informative features get fixated more frequently or for longer can vary depending on the experimental paradigm, the method of analysis, and the comparison (a contrast across emotions for a given location or a contrast across locations, such as the eye and mouth regions, for a given emotion). Nonetheless, the findings include more time fixating the eye region (in some cases taken to include the eyebrows, in some cases not) for fearful or surprised faces or both (Duran & Atkinson, 2021; Guo, 2012; Schurgin et al., 2014; Sullivan, Ruffman, & Hutton, 2007) and sad faces (Beaudry, Roy-Charland, Perron, Cormier, & Tapp, 2014; Eisenbarth & Alpers, 2011); more time fixating the mouth of happy faces (Beaudry et al., 2014; Eisenbarth & Alpers, 2011; Scheller, Büchel, & Gamer, 2012; Yitzhak, Pertzov, Guy, & Aviezer, 2022) and the mouth of fearful and surprised faces when both these emotions are presented within a block of trials (Duran & Atkinson, 2021); more time fixating on or very near the nose (Duran & Atkinson, 2021; Guo, 2012) or upper lip (Poncet et al., 2021; Schurgin et al., 2014) and other parts of the mouth (Duran & Atkinson, 2021; Poncet et al., 2021; Yitzhak et al., 2022) of disgusted faces; and more time fixating the central brow of angry faces (Duran & Atkinson, 2021; Poncet et al., 2021). Furthermore, one study found that misclassifications of facially expressed emotions were associated with fixations not only on emotion-informative facial regions but also on less relevant facial regions containing ambiguous cues or cues relevant to other emotional expressions (Poncet et al., 2021).

Most of these previous studies (with the notable exception of Poncet et al., 2021) rely on region-of-interest (ROI) analyses, which have several limitations, including variability and a lack of objectivity in the size, shape, and location of the ROIs (for discussion and some alternatives, see, e.g., Chuk, Chan, & Hsiao, 2014; Fuhl et al., 2018; Hessels, Kemner, van den Boomen, & Hooge, 2016; Orquin, Ashby, & Clarke, 2016; Paparelli et al., 2024; Rim, Choe, Scrivner, & Berman, 2021). Here, we have taken a different approach that does not rely on ROI analyses. To establish whether people preferentially fixate emotion-informative facial features when deciding upon the expressed emotion, we tested whether the patterns of fixations across the whole face can predict the viewed emotion. Further, we sought to understand the differential roles of foveal and extrafoveal vision in determining the location and spatiotemporal organization of those fixations, as discussed next.

### What are the distinct contributions of foveal and extrafoveal vision to determining where people direct their gaze on faces during emotion judgments?

Elimination of foveal vision in the blindspot and neutral blindspot conditions of our experiment removes the possibility for observers to extract the highest fidelity visual information about emotion-informative features, thus forcing a greater reliance on the coarser information provided by extrafoveal vision. In these two experimental conditions, then, fixations might follow one of three patterns. First, fixations might cluster more centrally on the face than in the unrestricted condition, reflecting a more global information processing strategy. This prediction is based on previous evidence suggesting that fixations at face center are indicative of and encourage a global information-processing strategy (Blais et al., 2008; Caldara et al., 2010; Miellet, Caldara, & Schyns, 2011), in which face perception task performance relies on extracting information about combinations of or relations between features (i.e., configural information), thus relying on extrafoveal visual processing. This contrasts with a more local information-processing strategy, which is characterized by foveal processing of individual facial features (Blais et al., 2008; Caldara et al., 2010; Miellet et al., 2011). Second, fixations in the blindspot and neutral blindspot conditions might instead cluster around rather than on emotion-informative facial features, thus maximizing the fidelity of the visual information about those features available to observers. Third, the clustering of fixations in these two conditions might show no clear differences compared with the clustering of fixations in the unrestricted condition.

We thus expected to find clusters of fixations on emotion-informative facial features, especially in the unrestricted condition, with possible variations on this pattern for the blindspot and neutral blindspot conditions, as noted in the previous paragraph. Elimination of extrafoveal vision in the spotlight condition, by contrast, eliminates the possibility of low-level visually salient or higher level features of the faces to guide eye movements, forcing our observers to rely on top–down knowledge of facial structure and of emotion-informative facial features to determine where they fixate. Thus, any clustering of fixations on emotion-informative facial features in the spotlight condition will indicate that observers possess and use knowledge of the emotion-informativeness of those features to guide their eye movements. Existing evidence suggestive of such a goal-driven influence on eye-gaze patterns comes from two studies that found a tendency for fixations to cluster on some emotion-informative facial regions even when viewers sought evidence of emotion within neutral faces (Schurgin et al., 2014; Vaidya, Jin, & Fellows, 2014).

## Methods

**Figure 1.**
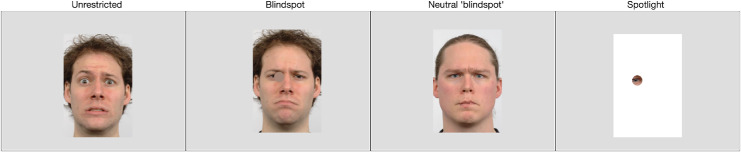
Frames from examples of each of the four stimulus presentation conditions. In the blindspot, neutral blindspot, and spotlight conditions, the circular mask or aperture subtended 2° visual angle and was presented gaze contingently, centered at the point of fixation. The gaze-contingent mask in the neutral blindspot condition (in this image, located on the mouth) was the equivalent part of the face from the neutral expression image of the same individual. Face images are reproduced, with permission and minor amendment, from the Radboud Faces Database (http://www.rafd.nl) (Langner et al., 2010).

Participants classified the emotion displayed on static images of faces under four presentation conditions (see Figure 1). In the control condition, participants viewed the faces unaltered, without gaze-contingent presentation. In the blindspot condition, foveal visual processing of the face was prevented by masking the fixated region with an opaque gray circle, subtending 2° visual angle, in a gaze-contingent fashion. The neutral expression blindspot condition was like the standard blindspot condition, but here the foveated region was dynamically updated, with the corresponding part of the face image of the same identity showing a neutral expression instead of with an opaque gray circle; thus, foveal vision received the relevant part of a neutral face whereas extrafoveal vision received the corresponding emotional face. Finally, in the spotlight condition, all extrafoveal regions were blocked by an opaque gray screen, allowing only foveal processing of the face. In this latter condition, the region of the image outside of the aperture was colored white, thus giving the participants a reference frame for the location of the face. This was important because the location of the image varied across trials (see Design and procedure section for details).

### Transparency and openness

We report how we determined our sample size, all data exclusions (if any), all manipulations, and all measures in the study, and the study follows Journal Article Reporting Standards (Appelbaum et al., 2018). All data, analysis code, and research materials are available at https://osf.io/kywuc/. Data were analyzed using JASP 0.18.13 (https://jasp-stats.org/) and toolboxes and custom code in MATLAB R2023b (https://www.mathworks.com), as detailed below. This design of the study and its analysis were not pre-registered.

### Participants

Participants were recruited from the Department of Psychology, Durham University, undergraduate participant pool, who were compensated with course credit, and by word of mouth from the wider undergraduate population at Durham University. A total of 44 participants were initially recruited; however, the data for nine of these participants were excluded due to problems with eye-tracking calibration (six participants) or incomplete eye-tracking data (three participants each had one whole block of missing eye-tracking data). Complete emotion classification and eye-tracking data were obtained and analyzed for 35 participants (28 female, seven male; ages 18 to 22 years; 30 of European and five of East Asian ethnicities).

We did not perform a priori power analyses to estimate our desired sample size. We therefore conducted sensitivity analyses using G*Power (Faul, Erdfelder, Buchner, & Lang, 2009) to establish the minimum effect sizes of interest given our sample size of 35, α = 0.05, and 80% power. Our primary analyses were repeated-measures analyses of variance (ANOVAs) and paired-sample and one-sample *t*-tests. A sensitivity analysis for a within-subjects main effect with four levels (corresponding to our main effect of stimulus presentation condition, as detailed below) revealed minimum detectable effect sizes of partial eta squared (η*_p_*^2^) = 0.1 without sphericity correction and η*_p_*^2^ = 0.138 with sphericity correction ε = 0.6. An additional sensitivity analysis for a within-subjects main effect with five levels (corresponding to our main effect of emotion, as detailed below) showed minimum detectable effect sizes of η*_p_*^2^ = 0.083 without sphericity correction and η*_p_*^2^ = 0.101 with sphericity correction ε = 0.7. For a paired-samples, two-tailed *t*-test, a sensitivity analysis showed a minimum detectable effect size of Cohen's *d_z_* = 0.429. For a one-sample, two-tailed *t*-test, a sensitivity analysis showed a minimum detectable effect size of Cohen's *d* = 0.487. Any observed effect sizes for our analyses with values less than these corresponding minimum detectable effect sizes are therefore to be regarded with caution (we highlight any such cases in the Results).

All participants had normal or corrected-to-normal vision. Those with corrected vision wore contact lenses. The study was conducted with approval from Durham University's Department of Psychology Ethics Committee. All participants gave informed consent prior to their participation. Data were collected between December 2021 and February 2022.

### Stimuli and apparatus

Face images were selected from the Radboud Faces Database (http://www.rafd.nl) (Langner et al., 2010). All faces were of Caucasian adults with full frontal pose and gaze. The selected stimulus set was comprised of 60 images: 12 individual identities (six male and six female),^3^ in each of five emotions: anger, fear, surprise, disgust, and sadness.^4^ The facial images were displayed in color and were cropped so that the image would take up a larger proportion of the viewing screen; the presented size was 384 (width) × 576 (height) pixels.

Stimuli were presented on a cathode-ray tube (CRT) monitor with a viewable screen size of 361 mm (width) × 271 mm (height), a resolution of 1024 (width) × 768 (height) pixels, and a refresh rate of 85 Hz. The participants were seated on a height-adjustable chair directly in front of the monitor with their head position controlled by a head and chin rest such that the viewing distance from the monitor screen was 50 cm. At this viewing distance, the face within the image subtended approximately 14° to 18° visual angle vertically from top of forehead to bottom of chin (depending on the expressor and expression). To measure binocular eye movements and to control the presentation of gaze-contingent stimuli, an EyeLink 1000 video-based eye tracker (SR Research, Ottawa, ON, Canada) with a sampling rate of 1000 Hz was used in a small, darkened room. The eye tracker was calibrated to the participants’ right and left eyes 24 times throughout the duration of the experiment (at the beginning of each stimulus presentation condition and every 11 trials within each condition), using a five-point manual calibration. A five-point calibration was chosen because it best represented the positions in which the face stimulus would be presented on the monitor screen and was most time efficient given the large number of calibrations to be done for each participant. For data analyses, the default criteria for fixations, blinks, and saccades implemented in the EyeLink system were used, except that we used a minimum duration for fixations of 100 ms. The experiment was executed and controlled using custom code in MATLAB and Psychophysics Toolbox Version 3 (Brainard, 1997) functions.

### Design and procedure

A within-participants design was implemented, with two repeated-measures variables: emotional expression (five levels: anger, disgust, fear, surprise, sadness) and stimulus presentation condition (four levels). The four stimulus presentation conditions were an unrestricted viewing condition (no gaze-contingent manipulation); a gaze-contingent central blindspot (no face information in foveal vision, relevant emotion in extrafoveal vision); a gaze-contingent neutral blindspot (relevant emotion in extrafoveal vision, neutral expression in foveal vision); and a gaze-contingent central spotlight (relevant emotion in foveal vision, no face information in extrafoveal vision).

All 60 face images (12 identities × 5 emotions) were presented once in each of the stimulus presentation conditions, in a new random order for each condition and participant. Thus, participants received a total of 240 image presentations (four repetitions of each of the 60 unique images). The order of stimulus presentation conditions was counterbalanced across participants. (An examination of stimulus presentation condition order effects on task performance and patterns of fixation is presented in the Supplementary Materials.)

For each of the four stimulus presentation conditions, the participant was first presented with a fixation cross at or near the center of the screen, which, when fixated, would allow the face to be presented. The fixation cross could appear at any one of 25 locations, which were randomly allocated across trials, and the subsequent face image was presented centered on this fixation cross. This resulted in the exact screen location of the fixation cross, and thus the exact location of the features (e.g., an eye, the mouth) of the subsequently presented face, being unpredictable. The fixation cross locations were offset from screen center by 0, 25, or 50 pixels left or right and up or down. The fixation-contingent onset of the face required participants to fixate within 30 pixels (1.2° of visual angle) of the center of the fixation cross for six consecutive eye-tracking samples for the face presentation to be triggered. A face image then immediately appeared on the screen for 3 seconds, during which time the participant viewed the face under the instruction to decide what emotion was displayed on the face. The 3-second presentation time was chosen based on findings from Bindemann et al. (2009) that this duration was sufficient to capture fixations across different features of frontal faces, including the mouth, which tended to be fixated later than other features (between 1250 and 2050 ms from stimulus onset). Participants provided their response after face offset by pressing one of the assigned response keys from the number keys along the top of the computer keyboard: 1 for anger, 2 for fear, 8 for surprise, 9 for disgust, and 0 for sadness. A response screen detailing these numbers appeared after the face image so that the participants knew how to respond. A calibration of the eye tracker was implemented every 11 trials to ensure accurate tracking of the participant's eye movements. Between each block of trials (stimulus presentation condition) the participant was given a rest break, if required, and once again underwent calibration prior to beginning the next block.

### Data analyses

#### Emotion classification response accuracy and response time

Our primary measure of emotion classification performance was accuracy, assessed using unbiased hit rates (Wagner, 1993),^5^ calculated for each emotion and stimulus presentation condition. The unbiased hit rate was chosen over standard hit rates because the frequency with which participants used the different response labels in each experiment was not equal (as reported in the Results). We also report, in the Supplementary Materials, the same set of analyses using hit rates (proportion correct values). These two sets of accuracy analyses produced very similar results. We also report an analysis of response times for correct responses, as a secondary measure of emotion classification performance. For this analysis, trials with extreme outliers, defined as response times that fell outside of 5 *SD* from the participant's overall mean correct response time, were removed prior to the analysis. These accounted for 23/6981 = 0.33% of all trials with correct responses (all of which were very slow response times that were greater than the mean + 5 *SD*). No participants were excluded from the analyses because of this outlier trial removal.

Repeated-measures ANOVAs were conducted separately for each of these accuracy and response time measures, with the within-participant variables stimulus presentation condition (unrestricted, blindspot, neutral blindspot, spotlight) and emotion (anger, fear, disgust, sadness, surprise). Statistical analyses were performed in JASP. Greenhouse–Geisser correction for degrees of freedom was used for all ANOVA main effects and interactions for which Mauchly's test of sphericity had *p* < 0.05. All post hoc tests were corrected for the relevant number of multiple comparisons using the Bonferroni–Holm method (uncorrected *p*-values are reported). Where the data failed to meet the normality assumption for *t*-tests (as indicated by Shapiro–Wilk tests, *p* < 0.05), non-parametric Wilcoxon signed ranks tests were used. For ANOVAs, we report the η*_p_*^2^ measure of effect size; for *t*-tests and Wilcoxon tests, we report Cohen's *d_z_* effect sizes or matched-rank biserial correlations, respectively, with 95% confidence intervals (CIs), as output by JASP.

#### Gaze behavior: Gaze decoding

We used a classification technique developed by O'Connell and Walther (2015), implemented in MATLAB, to predict the viewed facial emotion category in each of the stimulus presentation conditions, and the stimulus presentation conditions themselves (collapsed over emotion category), from participants’ gaze patterns. This classification technique computes category predictions by comparing each participant's fixation density on a trial-by-trial basis to the average of the fixation density for each emotion category or presentation condition of all other participants. This procedure is then repeated for each participant in a leave-one-subject-out (LOSO) cross-validation. The procedure begins with the calculation of fixation density maps (FDMs) from the non–left-out participants’ fixation data. FDMs are the sums of these fixations as a function of their location (*x* and *y* coordinates), weighted by their duration. Category-specific FDMs are then created for each of the image categories by averaging over all trials of a given category for all participants except the one held out. These average category-specific maps are then smoothed with a two-dimensional Gaussian kernel (in the present case, smoothing was implemented with σ = 13 pixels, equivalent to 0.5° visual angle, reflecting the upper bound of the typical accuracy of the EyeLink 1000 eye tracker) and *z*-scored. Marginal FDMs were also created by subtracting the grand mean of all FDMs from each category-specific FDM, thus controlling for spatial biases common across all image categories, especially the center bias (e.g., Bindemann et al., 2009; Findlay, 1982; He & Kowler, 1989; Tatler, 2007).

To predict the experimental conditions from gaze patterns, the code from O'Connell and Walther (2015) compares the FDMs from individual trials to the category-specific marginal FDMs in order to generate a goodness-of-fit score for each category. The goodness-of-fit score is calculated separately for each category by summing the marginal fixation map values over the fixated locations on each trial, weighted by fixation duration. The category with the largest goodness-of-fit score is then selected as the predicted category for that trial. If the prediction matches the true category label for the trial, the prediction is correct, and incorrect if not. This procedure is then repeated for each participant. The prediction accuracy is computed as the fraction of trials with correct category predictions. These prediction accuracy scores, one for each participant, are then tested for significance using one-sided *t*-tests comparing the prediction accuracy to chance (1/number of categories). We report the means and distributions of these decoding classification accuracies and the results of the associated *t*-tests, and we provide heatmap visualizations of the FDMs and marginal FDMs averaged across all participants. For further details of the gaze decoding, including the mathematical formulae used in the computations, see O'Connell and Walther (2015) (see also Damiano & Walther, 2019; Damiano, Wilder, & Walther, 2019).

We calculated emotion-specific FDMs and marginal FDMs separately for (a) all fixations on all trials, (b) all fixations on correct trials only, (c) first fixations on all trials, and (d) first fixations on correct trials only. We did this separately for each stimulus presentation condition. We also calculated FDMs and marginal FDMs for each emotion category collapsed across stimulus presentation condition, separately for all fixations on incorrect trials only and correct trials only, and for each stimulus presentation condition collapsed over emotion category, separately for (a) all fixations on all trials, (b) all fixations on correct trials only, (c) first fixations on all trials, and (d) first fixations on correct trials only. We provide qualitative comparisons of FDMs and marginal FDMs based on visual inspection and quantitative comparisons of FDMs using two sets of correlation analyses, as explained below.

The first set of correlation analyses tested whether FDMs for the same emotion across different categories of fixations (e.g., angry faces for all fixations in all trials vs. angry faces for all fixations in only trials with correct responses) were more similar than the corresponding FDMs for different emotions (e.g., angry faces for all fixations in all trials vs. disgusted faces for all fixations in only trials with correct responses). Thus, for the emotion-specific FDMs for each of the four stimulus presentation conditions (shown in Figures 4 to 7, respectively), we computed and then compared within-emotion correlations with between-emotion correlations as follows. Within-emotion correlations were computed for individual participant-level FDMs of the same emotion category (e.g., anger with anger, fear with fear) calculated from (a) all fixations in all trials versus all fixations in only trials with correct responses (panel A with panel E in Figures 4 to 7), (b) all fixations in all trials versus first fixations in all trials (panel A with panel B), (c) first fixations in all trials versus first fixations in only trials with correct responses (panel B with panel F), and (d) all fixations in trials with correct responses only versus first fixations in only trials with correct responses (panel E with panel F). Similarly, between-emotion correlations were computed for individual participant-level FDMs across each combination of different emotion categories (e.g., anger with disgust, anger with fear, anger with surprise, anger with sadness) calculated across the same four comparisons. In all cases, the MATLAB function corr2 was used, which returns a single correlation coefficient between two two-dimensional (2D) arrays (in this case, between a pair of individual participant-level FDMs). Then, for each of these four comparisons, the within-emotion correlations per participant were averaged together to obtain a mean within-emotion correlation coefficient for each participant for each comparison, and the between-emotion correlations were averaged together to obtain a mean between-emotion correlation coefficient for each participant for each comparison. Then, separately for each of the four comparisons, these mean correlation values were Fisher *z*-transformed and compared with a paired-samples *t*-test to assess whether the within-emotion correlations were, on average, larger than the between-emotion correlations. Doing so tested the hypothesis that the FDMs are more similar within the same emotions than between different emotions.

The second set of correlation analyses involved computing, for each class of fixations (e.g., all fixations on all trials), within-emotion correlations across all pairwise combinations of the four stimulus presentation conditions. This allowed us to assess the degree of similarity of emotion-specific FDMs between the different modes of stimulus presentation (and thus as a function of the availability of task-relevant foveal and extrafoveal information).

#### Gaze behavior: Recurrence quantification analysis

To further explore differences and similarities in fixation patterns across stimulus presentation conditions and emotion categories, we used recurrence quantification analysis (RQA). RQA is a method of nonlinear data analysis which quantifies the number and duration of recurrences of a dynamical system—in this case, sequences of fixational eye movements, as detailed in Anderson, Bischof, Laidlaw, Risko, and Kingstone (2013). The following RQAs were conducted using MATLAB code described in Anderson et al. (2013) and downloaded from https://barlab.psych.ubc.ca/research/. This code requires the user to select a radius value, which is the maximum distance between two fixations for them to count as recurring. We set this value to 2.5° of visual angle (62 pixels). In other words, fixations within a trial that were located within 2.5° of each other were considered refixations, whereas those greater than 2.5° of each other were considered fixations at different locations. In the setup for this experiment, this angular distance encompasses the approximate width of an eye, width of the lower nose, and height of a wide-open mouth, and about two-thirds the width of the mouth. This distance is sufficient to differentiate fixations on each eye and on the mouth, central/lower nose, and central brow (i.e., a fixation on one of these features would not count as a refixation on one of its immediately neighboring features). For most of the images in our stimulus set, this distance is not sufficient to differentiate fixations on an eye from those on an eyebrow (except for a small number of the fearful and surprised faces). Note that this value (2.5°) is different from the angular diameter (2°) of the gaze-contingent masks and window used in the experiment. This is because the rationales for these two parameters are fundamentally different: The size of the gaze-contingent masks and window was based on a conventional boundary separating foveal and extrafoveal vision (see footnote 1), whereas the angular distance for the RQA was chosen based on consideration of the size of and distances between key facial features in the images.

The RQA for eye movements toolbox produces four outcome measures: recurrence, which measures how often observers refixate previously fixated image positions, as indicated by the percentage of recurrent fixations; center of recurrence mass (CORM), which indicates how close or far in time refixations tend to occur; determinism, which measures repeating sequences of fixations; and laminarity, which indicates that specific areas of a scene are repeatedly fixated. We calculated each of these measures for each participant for each of the 20 experimental conditions (4 presentation conditions × 5 emotions), and then, for each measure, subjected the resulting data to repeated-measures ANOVAs.

## Results

### Emotion classification performance

#### Accuracy

A multinomial test in JASP revealed differences in the overall frequencies with which participants used the different response labels (irrespective of whether they were correctly applied), *χ^2^*(4) = 70.3, *p* < 0.001. Participants selected “angry” (total = 1815 trials, 21.6%; participant *M* = 51.9, *SD* = 8.2), “surprised”’ (total = 1808 trials, 21.5%; participant *M* = 51.7, *SD* = 8.9), and “sad” (total = 1792 trials, 21.3%; participant *M* = 51.2, *SD* = 8.4) more often than they selected “fearful” (total = 1510 trials, 18.0%; participant *M* = 43.1, *SD* = 8.7) and “disgusted” (total = 1475 trials, 17.4%; participant *M* = 42.1, *SD* = 9.5). This result motivated us to use the unbiased hit rates as a measure of emotion classification accuracy instead of standard hit rates (see Methods, above). Very similar results were also obtained when the analyses were conducted on the proportion correct hit rates, as reported in the Supplementary Materials. Confusion matrices are also presented in the Supplementary Materials, to allow for comparison of patterns of misclassifications across the four stimulus presentation conditions.

**Figure 2.**
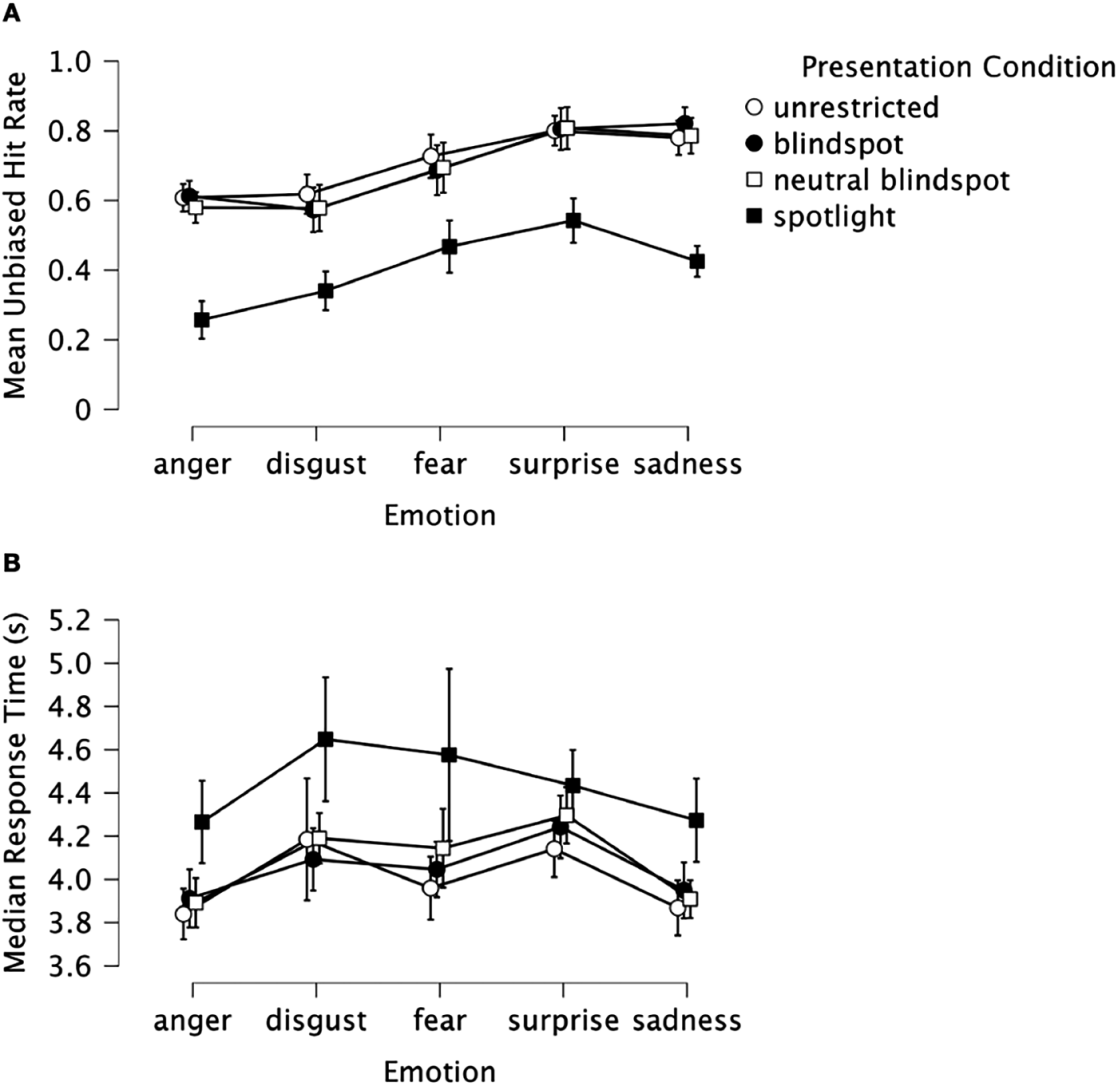
(**A**, **B**) Emotion classification accuracy (unbiased hit rates) (**A**) and median correct response times (**B**) as a function of emotion category and stimulus presentation condition. Circles and squares indicate mean values across participants; error bars indicate the 95% CIs.

The unbiased hit rates are summarized in Figure 2A. The ANOVA revealed significant main effects of stimulus presentation condition, *F*(3, 102) = 55.46, *p* < 0.001, η*_p_*^2^ = 0.66, and emotion, *F*(2.95, 100.43) = 31.57, *p* < 0.001, η*_p_*^2^ = 0.48, as well as a significant interaction, *F*(7.25, 246.48) = 2.86, *p* = 0.006, η*_p_*^2^ = 0.08. Simple main effects analyses revealed significant effects of stimulus presentation condition for all five emotions: anger, *F*(2.33, 79.35) = 79.73, *p* < 0.001, η*_p_*^2^ = 0.7; disgust, *F*(3, 102) = 25.13, *p* < 0.001, η*_p_*^2^ = 0.43; fear, *F*(3, 102) = 12.55, *p* < 0.001, η*_p_*^2^ = 0.27; surprise, *F*(3, 102) = 25.43, *p* < 0.001, η*_p_*^2^ = 0.43, and sadness, *F*(3, 102) = 75.52, *p* < 0.001, η*_p_*^2^ = 0.69. The η*_p_*^2^ values indicate larger effects of stimulus presentation condition for anger and sadness than for surprise, disgust, and especially fear. Paired-samples *t*-tests revealed, for all five emotions, reliably lower emotion classification accuracy for the spotlight condition compared with the other three stimulus presentation conditions (all *t* ≥ 4.42, all *p* < 0.001, minimum Bonferroni–Holm adjusted α = 0.005 for 10 comparisons). There were no other reliable differences in emotion classification accuracy across the stimulus presentation conditions for any of the five emotions (all |*t*| ≤ 1.75, all *p* ≥ 0.09).

#### Response time

We next conducted an analysis of times for correct responses (after removal of a small number of extreme outliers, as described in Methods). These data are summarized in Figure 2B. The presented and analyzed response time (RT) data are for 33 participants; the data for two participants were automatically excluded because they each had 0 correct trials in one of the 20 experimental conditions. A repeated-measures ANOVA on the median response times revealed significant main effects of stimulus presentation condition, *F*(2.23, 71.22) = 8.67, *p* < 0.001, η*_p_*^2^ = 0.21, and emotion, *F*(4, 128) = 14.21, *p* < 0.001, η*_p_*^2^ = 0.31, but no significant interaction, *F*(5.0, 159.94) = 1.19, *p* = 0.32, η*_p_*^2^ = 0.04. Although this was not a speeded response task and participants responded only after the face had disappeared from the screen after 3 seconds, participants were reliably slower to correctly classify the emotion in the spotlight condition (*M* = 4.44s, *SD* = 0.63) than in any of the other three stimulus presentation conditions: unrestricted (*M* = 4.0 seconds, *SD* = 0.39), *z*(32) = 3.8, *p* < 0.001, *r_rb_* = 0.76, 95% CI (0.54, 0.88); blindspot (*M* = 4.05 seconds, *SD* = 0.38), *z*(32) = 3.15, *p* = 0.001, *r_rb_* = 0.63, 95% CI (0.34, 0.81); and neutral blindspot (*M* = 4.09 seconds, *SD* = 0.34), *z*(32) = 3.06, *p* = 0.002, *r_rb_* = 0.61, 95% CI (0.31, 0.8). Correct response times did not differ significantly among the unrestricted, blindspot, and neutral blindspot conditions (all |*t*| ≤ 1.22 or |*z*| ≤ 1.81, all *p* ≥ 0.07). The main effect of emotion reflected reliably faster responses to expressions of anger (*M* = 3.98s, *SD* = 0.29) and sadness (*M* = 4.0s, *SD* = 0.28) than to expressions of fear (*M* = 4.18s, *SD* = 0.4), surprise (*M* = 4.28s, *SD* = 0.41), and disgust (*M* = 4.28s, *SD* = 0.38) (all |*t*| ≥ 5.06 or |*z*| ≥ 3.19, *p* < 0.001). None of the other pairwise comparisons between emotions was significant (all |*t*| ≤ 1.72 or |*z*| ≤ 1.78, *p* ≥ 0.096).

### Gaze behavior

#### Gaze decoding

We used gaze pattern decoding with 34-fold LOSO cross validation to determine if the gaze patterns (fixation location weighted by fixation duration) could discriminate the viewed emotion category and, separately, the stimulus presentation condition. Classification accuracies were compared with chance accuracy (1/5 for emotion category, 1/4 for stimulus presentation condition) using one-tailed *t*-tests.

**Figure 3.**
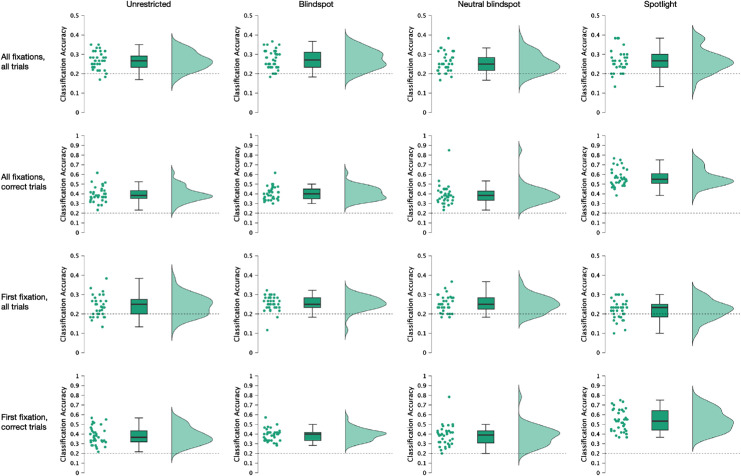
Classification accuracies for gaze decoding of the five emotion categories in the unrestricted (first column), blindspot (second column), neutral blindspot (third column), and spotlight (fourth column) stimulus presentation conditions, for all fixations on all trials (first row), all fixations on correct trials only (second row), first fixation on all trials (third row), and first fixation on correct trials only (fourth row). The dotted horizontal lines indicate chance classification performance. Individual dots indicate the mean classification accuracy scores for each participant. The distributions of these scores are shown on the far right of each plot. For the boxplots, the central horizontal line indicates the median score across participants; the bottom and top edges of the box indicate the 25th and 75th percentiles, respectively; and the whiskers indicate ±1.5 × the interquartile range.

We first tested whether fixation patterns could discriminate the viewed emotion category, separately for each stimulus presentation condition. The results are presented in Figure 3 and Table 1. For all four stimulus presentation conditions, emotion category was indeed predicted from the fixation data, and at levels substantially greater than chance, whether this was based on all trials or only (and even more so) on those trials in which participants provided correct emotion classification responses. Remarkably, these results held even when the analyses were based on only the data from first fixations in each trial.

**Table 1.** Results of the gaze decoding to determine classification accuracies of experimental conditions from the fixation data. Mean values are percent correct prediction accuracy scores from the LOSO cross-validations.

	*M*	*SD*	*t*(34)	*p*	*d* (95% CI)
Emotion category—unrestricted only (chance = 20%)					
All fixations, all trials	26.5%	4.6	8.3	5.5 × 10^−10^	1.4 (1.0, ∞)
First fixations, all trials	24.4%	5.4	4.79	1.6 × 10^−5^	0.81 (0.48, ∞)
All fixations, correct trials	39.5%	7.8	14.69	1.4 × 10^−16^	2.48 (1.91, ∞)
First fixations, correct trials	37.9%	9.0	11.77	7.6 × 10^−14^	1.99 (1.5, ∞)
Emotion category—blindspot only (chance = 20%)					
All fixations, all trials	27.4%	4.9	8.96	9.0 × 10^−11^	1.51 (1.1, ∞)
First fixations, all trials	25.4%	3.9	8.3	5.5 × 10^−10^	1.4 (1.0, ∞)
All fixations, correct trials	40.7%	6.8	17.99	3.0 × 10^−19^	3.04 (2.36, ∞)
First fixations, correct trials	39.2%	6.2	18.49	1.3 × 10^−19^	3.13 (2.43, ∞)
Emotion category—neutral blindspot only (chance = 20%)					
All fixations, all trials	25.6%	5.0	6.61	7.1 × 10^−8^	1.12 (0.76, ∞)
First fixations, all trials	25.1%	4.4	6.87	3.3 × 10^−8^	1.16 (0.79, ∞)
All fixations, correct trials	39.0%	10.4	10.85	6.9 × 10^−13^	1.84 (1.37, ∞)
First fixations, correct trials	38.2%	10.8	10.01	5.7 × 10^−12^	1.69 (1.25, ∞)
Emotion category—spotlight only (chance = 20%)					
All fixations, all trials	26.6%	6.0	6.53	9.0 × 10^−8^	1.1 (0.74, ∞)
First fixations, all trials	22.0%	4.9	2.41	0.011	0.41 (0.11, ∞)
All fixations, correct trials	56.4%	9.0	23.97	3.5 × 10^−23^	4.05 (3.19, ∞)
First fixations, correct trials	53.8%	11.1	18.07	2.6 × 10^−19^	3.05 (2.38, ∞)
Stimulus presentation condition (chance = 25%)					
All fixations, all trials	40.4%	6.7	13.52	1.5 × 10^−15^	2.29 (1.74, ∞)
First fixations, all trials	35.8%	7.3	8.74	1.6 × 10^−10^	1.48 (1.07, ∞)
All fixations, correct trials	52.8%	5.5	29.97	2.5 × 10^−26^	5.07 (4.0, ∞)
First fixations, correct trials	49.0%	7.7	18.59	1.1 × 10^−19^	3.14 (2.45, ∞)

**Figure 4.**
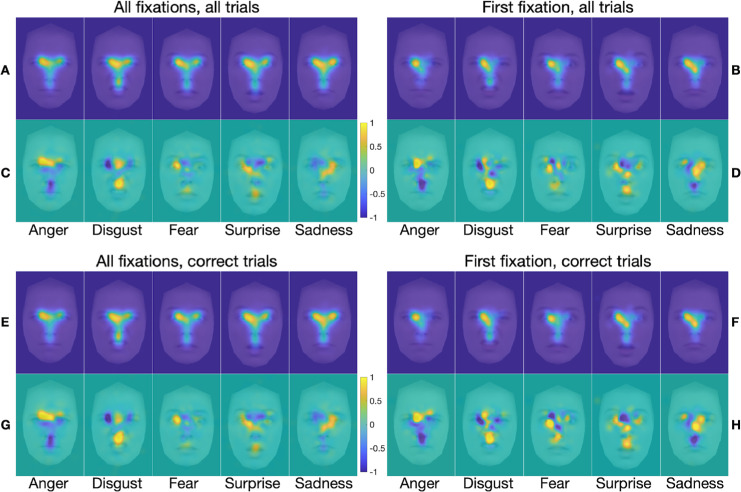
(**A**–**H**) FDMs (**A**, **B**, **E**, **F**) and marginal FDMs (**C**, **D**, **G**, **H**) for the five emotion categories in the *unrestricted* presentation condition only, for all fixations on all trials (**A**, **C**), first fixation on all trials (**B**, **D**), all fixations on correct trials only (**E**, **G**), and first fixation on correct trials only (**F**, **H**). Data are from all 35 participants, overlaid on a morphed average face for the relevant emotion. Note that maps for the gaze pattern decoding analysis were computed only from the 34 training subjects in each LOSO cross-validation fold (see Methods). The scale applies to the marginal FDMs, where positive values (warmer color) indicate regions with higher than average fixation density, and negative values (cooler color) represent regions with lower than average fixation density.

**Figure 5.**
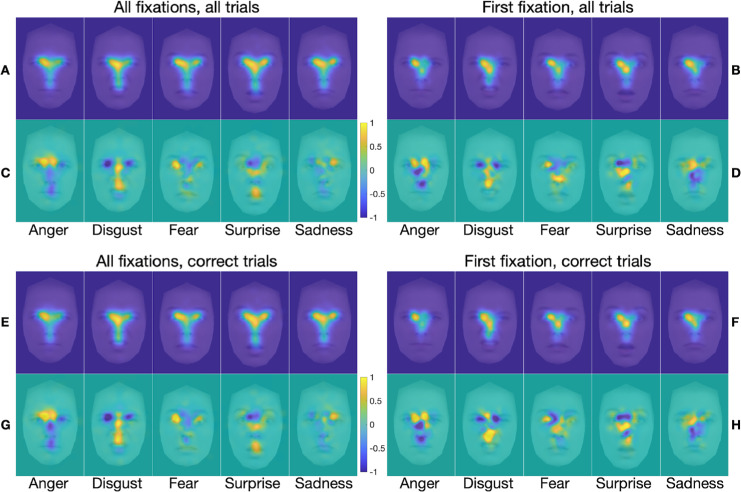
(**A**–**H**) FDMs (**A**, **B**, **E**, **F**) and marginal FDMs (**C**, **D**, **G**, **H**) for the five emotion categories in the blindspot stimulus presentation condition only, for all fixations on all trials (**A**, **C**), first fixation on all trials (**B**, **D**), all fixations on correct trials only (**E**, **G**), and first fixation on correct trials only (**F**, **H**). Additional figure legend details are as for Figure 4.

**Figure 6.**
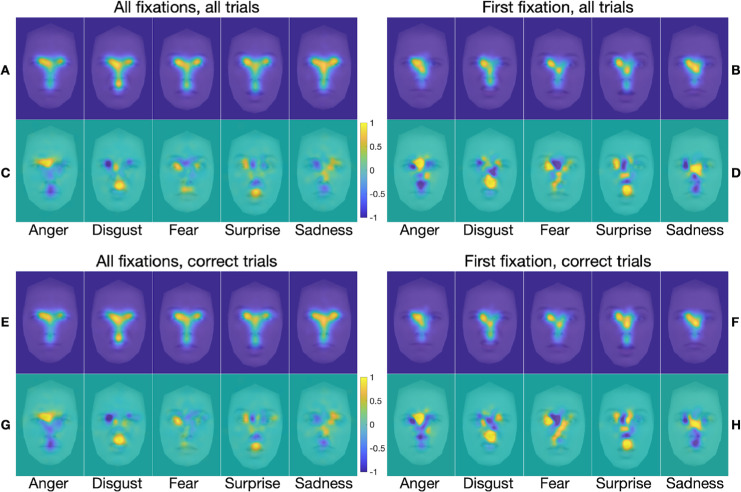
(**A**–**H**) FDMs (**A**, **B**, **E**, **F**) and marginal FDMs (**C**, **D**, **G**, **H**) for the five emotion categories in the neutral blindspot stimulus presentation condition only, for all fixations on all trials (**A**, **C**), first fixation on all trials (**B**, **D**), all fixations on correct trials only (**E**, **G**), and first fixation on correct trials only (**F**, **H**). Additional figure legend details are as for Figure 4.

**Figure 7.**
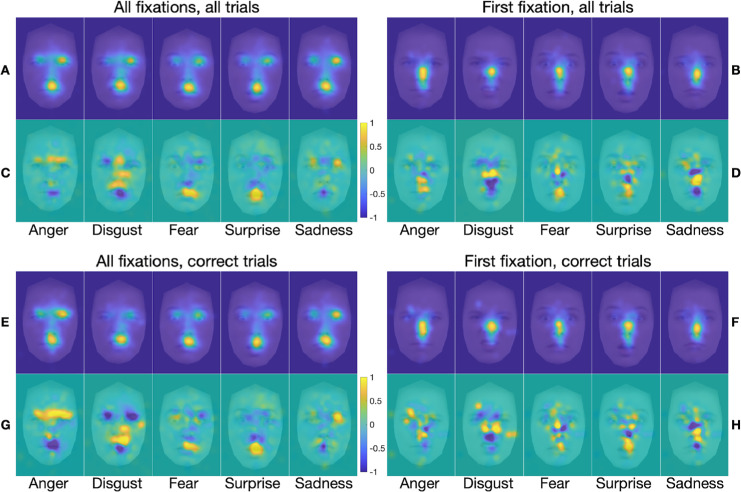
(**A**–**H**) FDMs (**A**, **B**, **E**, **F**) and marginal FDMs (**C**, **D**, **G**, **H**) for the five emotion categories in the *spotlight* stimulus presentation condition only, for all fixations on all trials (**A**, **C**), first fixation on all trials (**B**, **D**), all fixations on correct trials only (**E**, **G**), and first fixation on correct trials only (**F**, **H**). Additional figure legend details are as for Figure 4.

FDMs and marginal FDMs for each emotion category in each of the stimulus presentation conditions are presented in Figures 4 to 7. Each figure shows separate FDMs and marginal FDMs for all fixations and for first fixations, for all trials and for correct trials only. For the unrestricted presentation condition (Figure 4), the FDMs show clear commonalities in fixation patterns across emotions, with two patterns most evident. First, first fixations were concentrated mostly on or near the inner corner of the left eye, although they also included some spread to the nose region, especially for surprised and sad faces. This finding is broadly consistent with previous findings of first fixations to a location near face center, just below the eyes and slightly to the left of the face midline (Hsiao & Cottrell, 2008; Liu et al., 2024; Peterson & Eckstein, 2012). Second, across all fixations in a trial, fixations patterns clearly reflected the classic T-shaped distribution, with greatest concentration in the eyes, especially the left eye. Nonetheless, clear differences between emotions are also evident, especially in the marginal FDMs, in which commonalities in fixation density across image categories (such as the center bias) have been subtracted out. For angry expressions, there was greater fixation density at the central brow and inner eyebrows, especially on the left side. For disgusted faces, fixation density was highest at the philtrum, spanning the region of the lower nose and upper lip, and at the central brow, although for a smaller portion of the brow than for the angry faces. For fearful faces, fixation density was highest at the left eye and somewhat less so at the central upper lip area. For surprised faces, there was high fixation density at the left eye, spreading across to the mid nose, and at the central mouth. And, for sad faces, there was high fixation density at the inner right eye, spreading across and down to the lower nose. These emotion-specific patterns of highest fixation density line up well with previously identified emotion-diagnostic regions (e.g., Smith et al., 2005; Smith & Merlusca, 2014; Smith & Schyns, 2009). Any differences in the FDMs and marginal FDMs between all trials and correct trials only are more subtle.

It is also notable that the FDMs associated with the same emotions when calculated based on all fixations and on first fixations were more like each other than were the FDMs associated with different emotions when calculated based on all fixations and on first fixations. This was confirmed by correlation analyses. When based on fixations from all trials, within-emotion correlations between all-fixation FDMs and first-fixation FDMs (Figures 4A and 4B) were on average significantly larger than between-emotion correlations between all-fixation FDMs and first-fixation FDMs, *t*(34) = 9.7, *p* < 0.001 (within-emotion *M* = 0.73, *SD* = 0.095; between-emotion *M* = 0.677, *SD* = 0.098). Likewise, when based on trials with correct responses only, within-emotion correlations between all-fixation FDMs and first-fixation FDMs (Figures 4E and 4F) were on average significantly larger than between-emotion correlations between all-fixation FDMs and first-fixation FDMs, *t*(34) = 9.56, *p* < 0.001 (within-emotion *M* = 0.709, *SD* = 0.093; between-emotion *M* = 0.646, *SD* = 0.092). Correlation analyses also confirmed that, when based on all fixations, within-emotion correlations between all-trial FDMs and correct-only trial FDMs (panels A and E in Figure 4) were on average significantly larger than between-emotion correlations between all-trial FDMs and correct-only trial FDMs, *t*(34) = 18.79, *p* < 0.001 (within-emotion *M* = 0.988, *SD* = 0.01; between-emotion *M* = 0.878, *SD* = 0.049). Likewise, when based on first fixations only, within-emotion correlations between all-trial FDMs and correct-only trial FDMs (Figures 4B and 4F) were on average significantly larger than between-emotion correlations between all-trial FDMs and correct-only trial FDMs, *t*(34) = 16.4, *p* < 0.001 (within-emotion *M* = 0.968, *SD* = 0.028; between-emotion *M* = 0.744, *SD* = 0.1).

The FDMs and marginal FDMs for the blindspot and neutral blindspot conditions (Figures 5 and 6, respectively) are very similar to those for the unrestricted condition; any differences are subtle. The FDMs and marginal FDMs for the spotlight condition (Figure 7), by contrast, are different from those for the other three stimulus presentation conditions. Indeed, within-emotion correlations across the stimulus presentation conditions for a given class of fixations revealed that the emotion-specific FDMs were, on average, highly similar across the unrestricted, blindspot, and neutral blindspot stimulus presentation conditions, and less similar in the spotlight condition (see Table 2 and Supplementary Table S2). Yet, there is still clearly a bias for fixations to cluster on emotion-informative regions specific to the viewed emotion, similar to the other three conditions when all fixations are considered, but not so clearly for first fixations only. Further correlation analyses (reported in the Supplementary Materials) revealed that, as with the unrestricted stimulus presentation condition, in all the other three conditions the FDMs are more similar within the same emotions than between different emotions. This was the case when comparing across FDMs calculated based on all fixations with those calculated based on first fixations, for all trials (panels A and B in Figures 5 to 7) and for correct trials only (panels E and F in Figures 5 to 7). This was also the case when comparing across FDMs calculated based on fixations from all trials with those calculated based on correct-only trials, for all fixations (panels A and E in Figures 5 to 7) and for first fixations only (panels B and F in Figures 5 to 7).

**Table 2.** Correlation coefficients for comparisons of emotion-specific FDMs across the stimulus presentation conditions. Correlation coefficients were calculated separately for each participant for each within-emotion comparison (anger unrestricted with anger blindspot, disgust unrestricted with disgust blindspot, etc., for FDMs calculated from all fixations in all trials), and then averaged over all emotions. The coefficients presented in the table are these emotion-specific correlations averaged over participants. The results of paired-samples *t*-tests comparing these within-emotion FDM correlations across pairwise combinations of the four stimulus presentation conditions are presented in Supplementary Table S2.

	*M*	*SD*
All fixations, all trials		
Unrestricted vs. blindspot	0.84	0.084
Unrestricted vs. neutral blindspot	0.831	0.103
Unrestricted vs. spotlight	0.459	0.136
Blindspot vs. neutral blindspot	0.847	0.074
Blindspot vs. spotlight	0.477	0.128
Neutral blindspot vs. spotlight	0.519	0.146
All fixations, correct trials		
Unrestricted vs. blindspot	0.821	0.089
Unrestricted vs. neutral blindspot	0.806	0.121
Unrestricted vs. spotlight	0.414	0.136
Blindspot vs. neutral blindspot	0.822	0.091
Blindspot vs. spotlight	0.434	0.129
Neutral blindspot vs. spotlight	0.466	0.145
First fixations, all trials		
Unrestricted vs. blindspot	0.704	0.129
Unrestricted vs. neutral blindspot	0.707	0.134
Unrestricted vs. spotlight	0.335	0.185
Blindspot vs. neutral blindspot	0.731	0.11
Blindspot vs. spotlight	0.373	0.198
Neutral blindspot vs. spotlight	0.394	0.189
First fixations, correct trials		
Unrestricted vs. blindspot	0.667	0.134
Unrestricted vs. neutral blindspot	0.666	0.151
Unrestricted vs. spotlight	0.303	0.171
Blindspot vs. neutral blindspot	0.689	0.128
Blindspot vs. spotlight	0.323	0.181
Neutral blindspot vs. spotlight	0.342	0.182

Next, we used the same gaze decoding technique to ask whether fixation patterns only on *incorrect* trials could distinguish among experimental conditions. That is, we tested whether fixation patterns on trials in which participants misclassified the emotional expression predicted the experiment condition. We also produced FDMs and marginal FDMs to highlight any differences in gaze allocation across the face as a function of experimental condition during trials with incorrect responses. We focused on the prediction of emotion category for the data collapsed across stimulus presentation condition, on the assumption that the relative paucity of trials with incorrect responses would have provided insufficient numbers of fixations to perform separate decoding analyses for each stimulus presentation condition. We therefore also present, for comparison, a decoding analysis and associated FDMs and marginal FDMs for the prediction of emotion category collapsed across stimulus presentation condition for trials with correct responses only.

**Figure 8.**
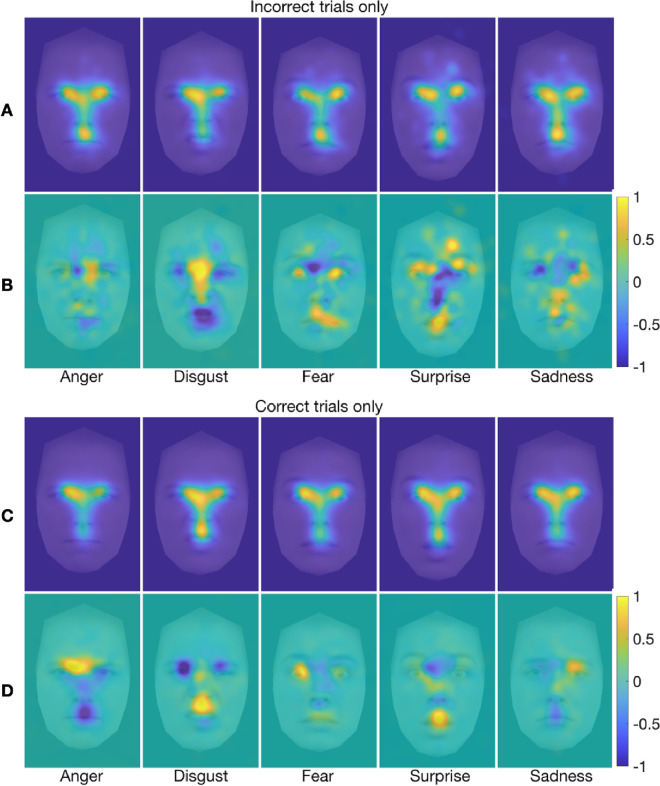
(**A**–**D**) FDMs (**A**, **C**) and marginal FDMs (**B**, **D**) for each emotion category (collapsed across stimulus presentation condition) for all fixations on incorrect trials only (**A**, **B**) and correct trials only (**C**, **D**), overlaid on an average face for the corresponding emotion. Additional figure legend details are as for Figure 4.

We found that fixation patterns on incorrect trials indeed predicted the expressed emotion on the face at levels substantially greater than chance (20%) when the data were collapsed across all stimulus presentation conditions, mean accuracy = 81.8%, *SD* = 5.22, *t*(34) = 69.98, *p* = 1.2 × 10^−38^, *d* = 11.83, 95% CI (9.37, ∞). Fixation patterns on correct trials also predicted the expressed emotion on the face at levels substantially greater than chance (when the data were collapsed across all stimulus presentation conditions), although only nearly half as accurately as for the incorrect trials, mean accuracy = 44.3%, *SD* = 5.4, *t*(34) = 26.59, *p* = 1.2 × 10^−24^, *d* = 4.5, 95% CI (3.54, ∞). FDMs and marginal FDMs for each emotion category collapsed across stimulus presentation condition are presented in Figure 8 for both incorrect and correct trials. There are clear differences in the FDMs and especially the marginal FDMs for the correct versus incorrect trials. These differences are consistent with the common confusions among certain emotions, particularly between anger and disgust, on the one hand, and between fear and surprise on the other (e.g., Calder et al., 2000; Jack et al., 2009; Schlosberg, 1941; Young, Perrett, Calder, Sprengelmeyer, & Ekman, 2002)—misclassifications also evidenced in the present study (see Supplementary Table S1). FDMs and marginal FDMs for all emotion-specific misclassifications (e.g., anger misclassified as disgust, as fear, as surprise, as sadness) are provided in Supplementary Figure S4.

**Figure 9.**
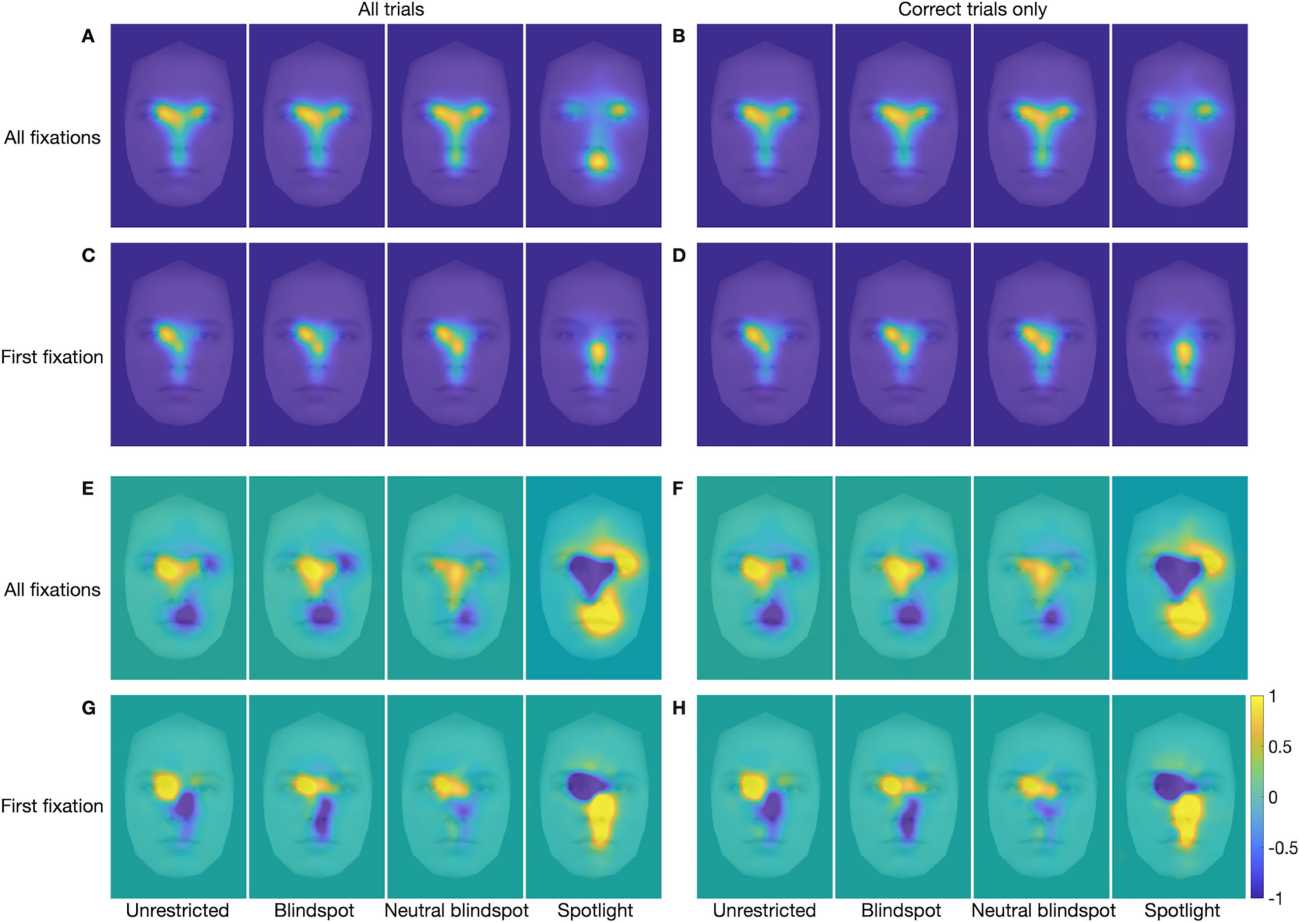
(**A**–**H**) FDMs (A–D) and marginal FDMs (**E**–**H**) for each stimulus presentation condition (collapsed over emotion category), for all trials (left-hand panels) and correct trials only (right-hand panels), for all fixations (panels **A**, **B**, **E**, **F**) and for first fixations only (**C**, **D**, **G**, **H**). Additional figure legend details are as for Figure 4.

Finally, we used the same gaze decoding technique to predict the stimulus presentation condition (collapsed over emotion category) from the fixation data. The results are presented in Table 1, and FDMs and marginal FDMs are presented in Figure 9 separately for all fixations and for first fixations, for all trials and for correct trials only. There is little noticeable difference in the FDMs and marginal FDMs for all trials versus correct trials only; the following description therefore applies to both. The FDMs for the unrestricted, blindspot, and neutral blindspot conditions are very similar to each other, with the greatest density of fixations located around the upper inner corner of the left eye, spreading toward the nasion/upper nose, followed by the same location above the inner corner of the right eye, with the remaining fixations spread along the nose via the philtrum to the top of the upper lip. The FDM for the spotlight condition, by contrast, is markedly different, with the greatest density of fixations at the philtrum/upper mouth, spreading to the lower nose, followed by the eyelid and upper region of the right eye, and only a relatively low density at the upper inner corner of the left eye, with very low or no fixation density on the upper nose. The marginal FDMs highlight the differences among the stimulus presentation conditions, controlling for commonalities across all conditions, such as the bias to fixate the image or face center. The blindspot and neutral blindspot marginal FDMs look almost identical, with greater fixation density than average in the area spanning the region from the upper inner corner of the left eye to the nasion/upper nose and lower fixation density than average in two locations: the region of the philtrum and right side of the upper lip, and the eyelid and upper outer region of the right eye. The marginal FDM for the neutral blindspot condition, by contrast, shows greater fixation density than average, mostly in the region of the nasion and upper nose, and on a smaller region of the upper inner corner of the left eye, with lower fixation density than average on the right side of the mouth and upper lip. The marginal FDM for the spotlight condition shows greater fixation density than average in two regions: one that covers most of the mouth and upper lip but especially the right side, spreading to the lower right of the nose, and the other on the outer half of the right eye and eyebrow, spreading to the central brow. The spotlight condition shows lower fixation density than average in a roughly triangular region spanning the inner eyes and nasion (with a bias to the left side) tapering down to the lower nose.

#### Recurrence quantification analysis

Having established that participants’ fixation patterns differed across facial emotion category and stimulus presentation condition, we next investigated how those fixation patterns changed over time. We used RQA to test whether the number and timing of recurrent fixations on the face images differed across experimental conditions. As noted in the Methods section, we defined recurrent fixations as fixations within a trial that were located within 2.5° visual angle of each other. In what follows, we detail the results of the four measures provided by this RQA: *recurrence*, which indicates how often observers refixate previously fixated image positions; *CORM*, which indicates how close or far in time refixations tend to occur; *determinism*, which indicates repeating sequences of fixations; and *laminarity*, which indicates that specific areas of a scene are repeatedly fixated. Here, we present the results for the data across all trials. For comparison, in the Supplementary Materials, we present the results for the data only from trials with correct emotion classification responses. The results for correct-only trials were broadly similar to those for all trials; any differences are noted below.

##### Recurrence

**Figure 10.**
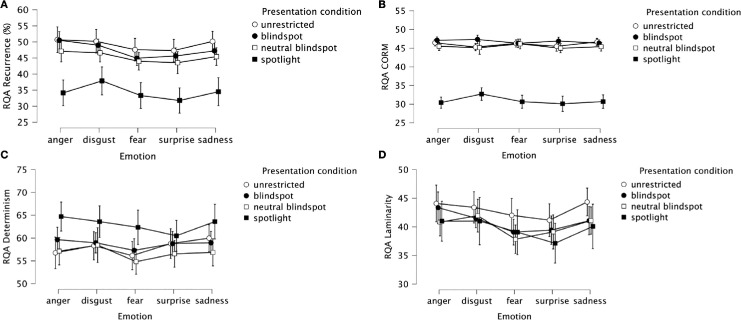
(**A**–**D**) RQA measures of recurrence (**A**), CORM (**B**), determinism (**C**), and laminarity (**D**), as a function of emotion and stimulus presentation condition. Circles and squares indicate mean values across participants; error bars indicate the 95% CIs.

The recurrence measures are summarized in Figure 10A. The repeated-measures ANOVA revealed significant main effects of stimulus presentation condition, *F*(2.03, 69.02) = 16.99, *p* < 0.001, η*_p_*^2^ = 0.33, and emotion, *F*(4, 136) = 12.59, *p* < 0.001, η*_p_*^2^ = 0.27. The interaction between stimulus condition and emotion was not significant, *F*(12, 408) = 1.4, *p* = 0.16, η*_p_*^2^ = 0.04. (For correct trials, the interaction was significant; see Supplementary Materials.) Pairwise comparisons to follow up the main effect of stimulus condition revealed the following. There were reliably lower recurrence values for the spotlight condition (*M* = 34.34, *SD* = 10.4) compared with the other three conditions: blindspot (*M* = 47.47, *SD* = 11.68), *t*(34) = 5.91, *p* < 0.001, *d_z_* *=* 1.0, 95% CI (0.59, 1.4); unrestricted (*M* = 49.21, *SD* = 13.61), *t*(34) = 4.91, *p* < 0.001, *d_z_* *=* 0.83, 95% CI (0.44, 1.21); and neutral blindspot (*M* = 45.35, *SD* = 13.51), *t*(34) = 3.92, *p* < 0.001, *d_z_* *=* 0.66, 95% CI (0.29, 1.03). In other words, participants refixated facial locations in the spotlight condition (in which extrafoveal information was absent) about 34% of the time, on average, which is considerably less often than the 45% to 49% refixations, on average, for the other conditions (in which extrafoveal information was present). The remaining pairwise comparisons were not significant (all *t* ≤ 1.9, uncorrected *p* ≥ 0.07).

Pairwise comparisons to follow up the main effect of emotion revealed the following. There were reliably higher recurrence values for disgusted faces (*M* = 45.94, *SD* = 9.34) compared with fearful faces (*M* = 42.46, *SD* = 9.03), *t*(34) = 5.69, *p* < 0.001, *d_z_* *=* 0.96, 95% CI (0.55, 1.34); surprised faces (*M* = 42.09, *SD* = 9.75), *t*(34) = 5.66, *p* < 0.001, *d_z_* *=* 0.96, 95% CI (0.55, 1.35); and sad faces (*M* = 44.34, *SD* = 9.72), *t*(34) = 2.89, *p* = 0.007, *d_z_* *=* 0.49, 95% CI (0.13, 0.84). There were also higher recurrence values for angry faces (*M* = 45.63, *SD* = 9.8) compared with fearful faces, *t*(34) = 3.93, *p* < 0.001, *d_z_* *=* 0.66, 95% CI (0.29, 1.03), and with surprised faces, *t*(34) = 4.15, *p* < 0.001, *d_z_* *=* 0.7, 95% CI (0.33, 1.07). Also, there were higher recurrence values for sad faces compared with surprised faces, *t*(34) = 3.17, *p* = 0.003, *d_z_* *=* 0.54, 95% CI (0.18, 0.89). Recurrence values were higher for sad faces compared with fearful faces, *t*(34) = 2.57, *p* = 0.015, *d_z_* *=* 0.43, 95% CI (0.08, 0.78), although this comparison did not survive correction for multiple comparisons (relevant Bonferroni–Holm adjusted α = 0.0125), and the effect size is effectively equal to the minimum detectable effect size of *d_z_* = 0.429 produced by our sensitivity analysis (see Methods). The remaining three pairwise comparisons were not significant (|*t*| ≤ 1.69, uncorrected *p* ≥ 0.1). In other words, participants refixated parts of fearful and surprised faces less often (about 42% of the time, on average) than they refixated parts of disgusted (∼46%), angry (∼45%), and sad (∼44%) faces.

##### Center of recurrent mass

The CORM measures are summarized in Figure 10B. The repeated-measures ANOVA revealed a significant main effect of stimulus presentation condition, *F*(1.81, 61.65) = 193.27, *p* < 0.001, η*_p_*^2^ = 0.85, and a significant interaction, *F*(7.42, 252.41) = 2.53, *p* = 0.014, η*_p_*^2^ = 0.07. The main effect of emotion was not significant, *F*(4, 136) = 1.05, *p* = 0.38, η*_p_*^2^ = 0.03. There were reliably and substantially smaller CORM values for the spotlight condition (*M* = 30.93, *SD* = 3.94) compared with the other three conditions: unrestricted (*M* = 46.04, *SD* = 3.1), *t*(34) = 16.84, *p* < 0.001, *d_z_ =* 2.85, 95% CI (2.09, 3.59); blindspot (*M* = 46.8, *SD* = 3.15), *t*(34) = 17.24, *p* < 0.001, *d_z_* *=* 2.91, 95% CI (2.14, 3.68); and neutral blindspot (*M* = 45.43, *SD* = 4.24), *t*(34) = 13.8, *p* < 0.001, *d_z_* *=* 2.33, 95% CI (1.68, 2.97). (All other |*t*| ≤ 2.25, uncorrected *p* ≥ 0.03.) In other words, when participants’ fixations returned to the same location, they tended to do so sooner in the spotlight condition than in the control, blindspot, or neutral blindspot conditions.

Simple main effects analyses to follow up the significant interaction revealed significant effects of emotion for the spotlight condition, *F*(4) = 3.42, *p* = 0.01, but not for the other stimulus presentation conditions (*F* ≤ 1.99, *p* ≥ 0.1). Pairwise comparisons for the spotlight condition revealed reliably larger CORM values for disgusted faces (*M* = 32.73, *SD* = 4.93) compared with angry faces (*M* = 30.43, *SD* = 4.34), *t*(34) = 3.32, *p* = 0.002, *d_z_* *=* 0.56, 95% CI (0.2, 0.91), and sad faces (*M* = 30.69, *SD* = 5.0), *t*(34) = 2.96, *p* = 0.0055, *d_z_* *=* 0.5, 95% CI (0.15, 0.85). CORM values were also larger for disgusted faces compared with surprised faces (*M* = 30.11, *SD* = 5.67), *t*(34) = 2.86, *p* = 0.007, *d_z_* *=* 0.48, 95% CI (0.13, 0.83), and fearful faces (*M* = 30.66, *SD* = 4.59), *t*(34) = 2.71, *p* = 0.01, *d_z_* *=* 0.46, 95% CI (0.11, 0.8); however, these comparisons did not survive correction for multiple comparisons (relevant Bonferroni–Holm adjusted α = 0.0063 and α = 0.0071, respectively). (All other |*t*| < 1, *p* ≥ 0.45.) In other words, in the spotlight condition only, participants’ refixations of facial regions tended to be further apart in time for disgusted faces than for angry and sad faces, and perhaps also for fearful and surprised faces (for disgusted faces, they returned to the same location later in time than they did for the other expression types).

##### Determinism

The determinism measures are summarized in Figure 10C. The repeated-measures ANOVA revealed significant main effects of stimulus presentation condition, *F*(2.2, 74.75) = 4.52, *p* = 0.012, η*_p_*^2^ = 0.12, and emotion, *F*(4, 136) = 2.54, *p* = 0.04, η*_p_*^2^ = 0.07. The interaction was not significant, *F*(12, 408) = 1.03, *p* = 0.4, η*_p_*^2^ = 0.03. Pairwise comparisons for the factor stimulus presentation condition revealed reliably larger determinism values for the spotlight condition (*M* = 62.96, *SD* = 8.42) compared with the neutral blindspot condition (*M* = 56.77, *SD* = 9.27), *t*(34) = 2.9, *p* = 0.007, *d_z_* *=* 0.49; 95% CI (0.14, 0.84). Determinism values were also larger for the spotlight condition compared with the blindspot condition (*M* = 58.74, *SD* = 6.89), *t*(34) = 2.7, *p* = 0.011, *d_z_* *=* 0.46, 95% CI (0.1, 0.8), and the unrestricted condition (*M* = 58.01, *SD* = 8.49), *t*(34) = 2.14, *p* = 0.039, *d_z_* *=* 0.36, 95% CI (0.02, 0.7), although these comparisons did not survive correction for multiple comparisons (relevant Bonferroni–Holm adjusted α = 0.01 and α = 0.0125, respectively), and the effect size for the latter comparison is less than the minimum detectable effect size of *d_z_* = 0.429 produced by our sensitivity analysis (see Methods). Pairwise comparisons for the factor emotion revealed smaller determinism values for fearful faces (*M* = 57.66, *SD* = 6.26) than for sad faces (*M* = 59.86, *SD* = 6.09), *t*(34) = 2.59, *p* = 0.014, *d_z_* = 0.44, 95% CI (0.09, 0.78), disgusted faces (*M* = 59.84, *SD* = 6.64), *t*(34) = 2.49, *p* = 0.018, *d_z_* = 0.42, 95% CI (0.07, 0.76), and angry faces (*M* = 59.57, *SD* = 5.58), *z*(34) = 1.97, *p* = 0.0495, *r_rb_* = 0.38, 95% CI (0.02, 0.65), although these comparisons did not survive correction for multiple comparisons (relevant Bonferroni–Holm adjusted α = 0.005, α = 0.0056, and α = 0.0063, respectively), and the effect size for the latter two comparisons is less than the minimum detectable effect size of *d_z_* = 0.429 produced by our sensitivity analysis (see Methods). (All other |*t*| ≤ 1.44, *p* ≥ 0.16.) In summary, the main finding from the determinism measure is that repeating sequences of fixations were more likely for the spotlight condition than for the other three stimulus presentation conditions, especially the neutral blindspot condition.

##### Laminarity

The laminarity measures are summarized in Figure 10D. The repeated-measures ANOVA revealed a significant main effect of emotion, *F*(4, 136) = 9.07, *p* < 0.001, η*_p_*^2^ = 0.21. The main effect of stimulus presentation condition and the interaction were not significant (*F* ≤ 1.14, *p* ≥ 0.33). The main effect of emotion reflected reliably smaller laminarity values for surprised faces (*M* = 39.22, *SD* = 8.62) and fearful faces (*M* = 39.51, *SD* = 8.41) compared with all three other emotional expressions: anger (*M* = 42.33, *SD* = 8.18) > surprise, *t*(34) = 3.94, *p* < 0.001, *d_z_* *=* 0.67, 95% CI (0.3, 1.03); anger > fear, *z*(34) = 3.39, *p* < 0.001, *r_rb_* = 0.66, 95% CI (0.39, 0.82); sadness (*M* = 41.66, *SD* = 8.38) > surprise, *t*(34) = 3.31, *p* = 0.002, *d_z_* *=* 0.56, 95% CI (0.2, 0.91); sadness > fear, *t*(34) = 3.13, *p* = 0.004, *d_z_* *=* 0.53, 95% CI (0.17, 0.88); disgust (*M* = 41.98, *SD* = 7.83) > surprise, *t*(34) = 3.56, *p* = 0.001, *d_z_* *=* 0.6, 95% CI (0.24, 0.96); disgust > fear, *t*(34) = 3.67, *p* < 0.001, *d_z_* *=* 0.62, 95% CI (0.25, 0.98). (All other |*t*| ≤ 1.03, *p* ≥ 0.3.) In other words, for fearful and surprised faces, specific sequences of fixations were less often repeated than for angry, disgusted, and sad faces.

In summary, we have established, using RQA, differences across some experimental conditions in the sequences of recurrent fixations. Where do these refixations occur, and how do the spatial distributions of those refixations differ across experimental conditions? It is reasonable to expect that the predominant patterns in the FDMs that we plotted for our experimental conditions (Figures 4 to 9) are determined by the refixations rather than by lone fixations (i.e., those that are not refixations). If so, then heatmaps of the refixations for each experimental condition should look very similar to the corresponding condition-specific FDMs. To verify this, we generated refixation heatmaps for each emotional expression category (collapsed over stimulus presentation conditions) to highlight those regions of the face that received the most refixations. These are reported in the Supplementary Materials. Note that these refixation maps differ from the FDMs (presented above) in that they represent the density of refixations without any weighting by fixation duration (rather than the density of all fixations weighted by their duration). Nonetheless, these emotion-specific refixation maps were indeed very similar to the FDMs and marginal FDMs shown in Figure 8, especially those for trials with correct responses.

## Discussion

We used a gaze-contingent paradigm to dynamically and independently manipulate the presence and absence of foveal and extrafoveal visual input when observers classified basic emotions (anger, disgust, fear, surprise, sadness) portrayed in faces. The stimuli were photographic images displayed so that the faces subtended a visual angle equivalent to viewing a real face at a close interpersonal distance, such that only a small part of the face (an area with a diameter approximately the width of the visible eyeball) would cover the fovea at any one time. We used this paradigm to address three questions: (a) What are the distinct contributions of foveal and extrafoveal visual processing to the ability to decipher what emotion a face is expressing? (b) When deciding what emotion a face is expressing, do people direct their gaze to the most informative facial features for the emotion or emotions in question? (c) What are the distinct contributions of foveal and extrafoveal vision to determining where people direct their gaze on faces (during emotion judgments)?

### What are the distinct contributions of foveal and extrafoveal visual processing to the ability to decipher what emotion a face is expressing?

Our first notable finding is that the absence of foveal information (in the blindspot condition) did not make any difference to accuracy or speed of emotion classification performance, compared with when both foveal and extrafoveal information was present (in the unrestricted condition). Previous research using gaze-contingent blindspots has similarly shown that foveal vision is not necessary but is beneficial for perceptual encoding during an object identification task (Henderson, McClure, Pierce, & Schrock, 1997) and for the detection and identification of animals in static scenes (Miellet, Zhou, He, Rodger, & Caldara, 2010), and is neither necessary nor beneficial for task performance in visual search for objects in real-world indoor and outdoor static scenes (Nuthmann, 2014). This contrasts with findings that gaze-contingent masking of foveal vision substantially disrupts the ability to read (Rayner & Bertera, 1979) and to locate a target letter in an alphanumeric array (Bertera & Rayner, 2000). Moreover, the absence of foveal information about specific features of emotionally expressive faces (in the neutral blindspot condition), as opposed to the absence of foveal information per se, also did not reduce emotion classification accuracy or speed. Extrafoveal information alone was sufficient for accurate and efficient emotion identification. As expected, there was a large drop in emotion classification performance when only foveal information was available (in the spotlight condition). This was the case for all five emotions tested, although for emotion classification accuracy the impact of removing extrafoveal information was greater for expressions of anger and sadness than it was for expressions of disgust, fear, and surprise. Previous research using gaze-contingent spotlights has similarly shown that restricting visual input to the foveal region dramatically impacts visual search performance for real-world static scenes (Nuthmann, 2014).

Our finding in the present study that foveal information about the emotional expression did not enhance emotion classification performance, compared with when only extrafoveal information about the face was available, is surprising when considered alongside our earlier findings from the enforced brief fixation paradigm. In that work, we found that foveation of certain emotion-distinguishing facial features enhanced emotion classification performance compared with foveation of less informative parts of the face and thus extrafoveal processing of the emotion-informative features (Atkinson & Smithson, 2020; Duran & Atkinson, 2021). We suggest two possible, but not necessarily mutually exclusive, reasons for these differences in findings. First, the extended sampling of extrafoveally presented emotion-informative regions in the blindspot and neutral blindspot conditions of the present study (compared with the single “snapshot” in the enforced fixation studies) might have provided sufficient information for the emotion judgment such that no additional benefit would have been derived from the addition of foveal processing of those emotion-informative regions in the unrestricted viewing condition. Second, the differences in findings might be partly explained by the operation of presaccadic attention in the present study, which is greatly reduced or absent in the enforced fixation paradigm used in our earlier studies. Presaccadic attention is the shift in visual attention to the location of an upcoming saccade before the onset of that saccade, which serves to prioritize and enhance visual processing at the upcoming fixation location (e.g., Deubel & Schneider, 1996; Kowler, Anderson, Dosher, & Blaser, 1995), and which includes, notably, the enhancement of high spatial frequency information (Kroell & Rolfs, 2021; Li, Barbot, & Carrasco, 2016; Li, Pan, & Carrasco, 2019). The paradigm of the present study affords multiple saccades and fixations on the face, thus allowing (thanks to presaccadic attention) the extraction of higher spatial frequencies from the saccade target locations than could be extracted from other extrafoveal locations on the face. In the enforced fixation paradigm, by contrast, the very brief duration of face presentation severely limited the planning and execution of saccades (to a maximum of one, and often none), thus greatly limiting the operation of presaccadic attention.

### When deciding what emotion a face is expressing, do people direct their gaze to the most informative facial features for the emotion or emotions in question?

The short answer to this second research question, as indicated by our results, is “yes, although not exclusively.” Our gaze decoding analyses revealed that facial emotion biased spatial attention such that the emotion category of individual face images was predicted from evoked gaze patterns. The associated marginal fixation density maps show that this bias of gaze allocation was toward emotion-informative facial features. With both foveal and extrafoveal information available (i.e., in the unrestricted condition, shown in Figure 4), there was relatively higher fixation density on the central brow and inner eyebrows, especially on the left side, for angry faces; the nasion/central brow and the philtrum (lower nose to upper mouth) for disgusted faces; the left eye and (less so) the upper mouth for fearful faces; the upper mouth for surprised faces (more so than for fearful faces) and an area spanning the left eye to the middle of the nose; and an area spanning the right eye to the mid-to-lower nose for sad faces. These emotion-specific biases in gaze allocation reflect the locations of some of the previously identified emotion-informative facial features in the mid- to high spatial frequency bands: the brow and eyebrows for anger, the nose and mouth and nasion for disgust, the eyes for fear, the mouth for surprise, and the right eye for sadness (Smith et al., 2005; Smith & Merlusca, 2014; Smith & Schyns, 2009). Not all emotion-informative facial features in the mid- to high spatial frequency bands attracted higher fixation densities than average, however—specifically, the eyes of angry faces, the mouth and the creases between the nose and cheeks of disgusted faces, and the (downturned) corners of the mouth and the inner eyebrows of sad faces.

Note that these findings were obtained using analytical methods (the gaze decoding analysis of O'Connell & Walther, 2015) that have not previously been used to examine gaze allocation within faces and do not suffer from the limitations of ROI analyses (as discussed in, for example, Chuk et al., 2014; Fuhl et al., 2018; Hessels et al., 2016; Orquin et al., 2016; Paparelli et al., 2024; Rim et al., 2021). Despite our different analytical methods, our findings show similarities with the findings of previous studies that also used unmanipulated face images but different analytical strategies to examine gaze behavior to emotion-informative features during emotion judgments. Our own earlier study (Duran & Atkinson, 2021), for example, reported that observers spent more time fixating the central brow for angry and disgusted faces than for fearful and surprised faces; the nose for disgusted faces than for fearful, surprised, and angry faces; the eyes for fearful and surprised faces than for angry and disgusted faces; and the mouth for fearful and surprised faces than for angry faces and for disgusted faces than for angry, fearful, and surprised faces. Those results were obtained using a ROI analysis in which the eyes, eyebrows, nose, mouth, and central brow were delineated as distinct ROIs. Also using a ROI analysis, but with just the eyes, nose, and mouth as ROIs, Guo (2012) reported that observers spent more time fixating the eyes of fearful, surprised, and angry faces than of happy, sad, and disgusted faces (where the eye ROI included the eyebrows but not the central brow), and more time fixating the mouth of surprised faces than of angry, disgusted, sad, and fearful faces. (For related findings, see also Beaudry et al., 2014; Schurgin et al., 2014; Wells, Gillespie, & Rotshtein, 2016.)

Thus, we provide evidence indicating that, when deciding what emotion a face is expressing, peoples’ gaze is biased to the most informative facial features for the emotion or emotions in question. Yet, we also found evidence that, when misclassifying facially expressed emotions, peoples’ gaze is also biased to features informative of the selected (incorrect) emotion. This can be seen in the marginal FDMs for correct and incorrect responses in Figure 8, which shows the differences in fixation distributions for each emotion type collapsed over the different misclassifications, and in Supplementary Figure S4, which shows the differences in fixation distributions for each specific misclassification (in both cases, these fixation densities are collapsed over the four stimulus presentation conditions).

For angry faces, trials with correct responses were characterized by a fixation bias to the anger-informative central brow and (mostly left) eyebrow region. In incorrect-response trials overall, by contrast, this fixation bias was less clear and more spatially focused to the nasion; there were also fixations in the philtrum (between the lower nose and upper lip). Angry faces were most often misclassified as sad (10%–28.3% of trials, depending on the stimulus presentation condition; see the confusion matrices in the Supplementary Materials), and such trials were associated with a lower than average fixation density in the anger-informative central brow and eyebrow region, with fixations concentrated instead on the upper nose, right eye, and mouth. The next most common misclassification of angry faces was as disgusted (4.5%–10.2% of trials), and such misclassifications were associated with fixations to the disgust-informative philtrum/lower nose region, but so were misclassifications of anger as fear or sad, although not as surprise.

For disgusted faces, trials with correct responses were characterized by a fixation bias primarily to the region of the philtrum and lower nose. Participants misclassified disgusted faces as angry much more frequently than as one of the other emotions (26.2%–38.3% of trials, on average, compared with a maximum of 4%) and more frequently than the converse misclassification—that is, misclassifications of angry faces as disgusted (4.5%–10.2%). Trials in which participants misclassified disgust as anger were characterized by a fixation bias to the eyes, eyebrows, and nasion and (to a lesser extent) to the mouth. Overall, across all misclassifications of disgusted faces, there was a fixation bias to the nasion and central brow.

For fearful and surprised faces, trials with incorrect responses overall showed quite similar fixation biases, notably including clusters on both the eyes and the mouth, yet correct responses for fearful faces were associated with a fixation bias primarily to the left eye, whereas correct responses for surprised faces were associated with a fixation bias primarily to the mouth. Fearful and surprised faces were sometimes confused with each other, with fearful faces misclassified as surprised (12.1%–21% of trials) more often than surprised faces were misclassified as fearful (3.3%–4.5% of trials for the unrestricted, blindspot, and neutral blindspot conditions; 16.2% of trials for the spotlight condition), and more often than these expressions were misclassified as angry, disgusted, or sad. Fearful faces misclassified as surprised were associated with higher than average fixation densities at the eyes, particularly the right eye, and the upper nose, and lower than average fixation density at the mouth. Surprised faces misclassified as fearful were associated with higher than average fixation densities, mostly at the mouth and the right eye, and lower than average fixation density at the central brow.

Finally, for sad faces correct trials were associated with a fixation bias to the right eyelid/eye, whereas incorrect trials overall were associated with fixation biases to the right eye, lower nose, and upper lip/philtrum. Errors for sad faces tended to involve misclassifications as disgust (3.3%–7.6% of trials), fear (2.9%–8.1% of trials), or anger (1.4%–14.0% of trials), but very rarely as surprise (0.2%–4.5% of trials). This pattern of misclassifications is more difficult to align with the fixation biases in trials with incorrect versus correct trials. Nonetheless, among the various regions highlighted in the marginal FDMs for misclassifications of sad faces it is notable that trials involving misclassifications as disgust or fear showed lower than average fixation densities in the very region associated with correct-response trials (i.e., the right eyelid/eye), and misclassifications as anger were associated with higher than average fixation densities in the lower but not upper region of the right eye.

In summary, these findings provide evidence that the spatial distribution of fixations is associated with both the correct and incorrect classification of emotional facial expressions (see also Poncet et al., 2021). Correct responses are associated with fixation biases to emotion-specific informative regions, whereas incorrect responses are associated with the absence of fixation biases to those emotion-specific informative regions and, in some cases, a fixation bias to a region informative of the (wrongly) labeled emotion.

The similarities between our results in the unrestricted presentation condition and those of Poncet et al. (2021) are notable, especially given that they also did not use ROI analyses. During a five-choice emotion classification task with expressions of anger, disgust, fear, sadness, and happiness, observers spent more time fixating an area encompassing the central brow, nasion, and inner eyebrows for angry faces, compared with the average for the other emotions tested; an area centered on the philtrum that extended from the lower nose to the mouth center and, separately and less strongly, the nasion/central brow of disgusted faces, compared with the average of the other emotions; an area extending from the lower portion of and just below the left eye across the nose to the lower portion of and just below the right eye (stronger on the left) for fearful faces, compared with the average of the other emotions; and both the left and right eyes, including small regions below the eyes, for sad faces, compared with the average of the other emotions. These findings were revealed using a method similar to, but nevertheless distinct from, the O'Connell and Walther (2015) method we used to construct the FDMs and marginal FDMs. (There are also some differences between the two studies in the stimulus materials and experimental design, which we shall not detail here.) In the study by Poncet et al. (2021), emotion-specific heatmaps were generated that represent the time the different areas of the face were within the 2°-diameter foveal zone of participants, averaged across participants and the different stimulus faces. They also constructed corresponding heatmaps that represent the results of pixelwise *t*-tests that compared the mean fixation time for a given emotion to the mean fixation time for the four other emotions. The similarity in the findings for expressions of anger, disgust, fear, and sadness across the two studies is notable not only because of the different analytical methods used but also because of the slightly different combination of emotions used (anger, disgust, fear, happiness, and sadness in the Poncet et al. study; anger, disgust, fear, surprise, and sadness in our study).

Our findings go beyond those reported by Poncet et al. (2021), in three important respects. First, we provide results for fixations on surprised faces, which show that participants spend more time looking at the left eye (as they do with fearful faces) and the central mouth (more so than for fearful faces), compared with the average. This latter result is consistent with our previous finding that an enforced single fixation on the center of the mouth allowed observers to more accurately distinguish between fearful and surprised expressions than did enforced fixation on certain other facial locations (Atkinson & Smithson, 2020; Duran & Atkinson, 2021), adding further support to the idea that fixating emotion-information regions—in this case, the mouth for expressions of surprise (Smith & Schyns, 2009; Smith et al., 2005)—aids emotion recognition performance and, particularly in this case, discrimination between fear and surprise.

The second respect in which our findings go beyond those reported by Poncet et al. (2021) is that we found the emotion-specific biases in gaze allocation to emotion-informative facial regions not only when aggregated across multiple fixations within trials (in our case, across the 3-second duration of image presentation) but also, remarkably, even for only first fixations within trials. This was the case not only for the unrestricted presentation condition (Figure 4) but also for the blindspot and neutral blindspot conditions (Figures 5 and 6, respectively), yet not for the spotlight condition (Figure 7). These results suggest that these emotion-informative features, projected to extrafoveal regions of the retina at the point of face onset (except in the spotlight condition), attract attention and thus gaze in a bottom–up fashion, presumably via sensory-driven aspects such as visual saliency and image features (e.g., Açık, Onat, Schumann, Einhäuser, & König, 2009; Itti & Koch, 2000; Kollmorgen, Nortmann, Schroder, & Konig, 2010; Veale, Hafed, & Yoshida, 2017). We discuss saccade target selection in more detail below.

The third respect in which our findings go beyond those reported by Poncet et al. (2021) is that, in addition to revealing emotion-specific gaze patterns when observers receive both foveal and extrafoveal inputs from the face, we have shown what happens to gaze patterns when either foveal or extrafoveal processing of the expression is prevented. We thus segue to consider our results in relation to the third of our research questions.

### What are the distinct contributions of foveal and extrafoveal vision to determining where people direct their gaze on faces (during emotion judgments)?

We found that the same emotion-specific gaze patterns as discussed above for the unrestricted viewing condition were evident even when we occluded foveal visual input (the blindspot condition; see Figure 5) or replaced foveal input of the expressive face with the corresponding region of the neutral face (the neutral blindspot condition; see Figure 6). In other words, what information did or did not project to the fovea did not in itself substantially alter the spatiotemporal distributions of fixations. This is consistent with the results of previous studies suggesting that the high-resolution information provided by foveal vision is not required for locating the target object within a real-world scene, as indicated by the presence, in both foveal blindspot and unrestricted conditions, of a preferred location for fixation on or near the center of target objects (Henderson et al., 1997; Miellet et al., 2010; Nuthmann, 2014). Nonetheless, there are also reports of differences in fixation times and eye-movement patterns as a result of gaze-contingent making of foveal vision, including in some of those same studies (e.g., Henderson et al., 1997). We found no evidence indicating that fixations in the blindspot and neutral blindspot conditions clustered more centrally on the face than in the unrestricted condition, which would have reflected the possible operation of a more global information processing strategy (as discussed in the Introduction; compare Figures 4, 5, and 6). Nor was there evidence that fixations clustered around rather than on the emotion-informative features in those two conditions, which would have reflected a compensatory strategy to maximize the fidelity of the visual information about those features.

Saccade target (fixation location) selection can be driven by bottom–up mechanisms based on sensory-driven aspects such as visual saliency and image features (as noted above) or on higher level features of the scene such as object identity or other semantic-level attributes (e.g., de Haas, Iakovidis, Schwarzkopf, & Gegenfurtner, 2019; Kiat, Hayes, Henderson, & Luck, 2022; Schütt, Rothkegel, Trukenbrod, Engbert, & Wichmann, 2019; Xu, Jiang, Wang, Kankanhalli, & Zhao, 2014), or by top–down mechanisms that are task specific or goal driven (e.g., Findlay & Gilchrist, 2003; Kollmorgen et al., 2010; Land, 2006; Yarbus, 1967). In the context of the present experiment, differences in eye movements and thus fixation patterns across emotion categories could be driven in a bottom–up fashion related to differences in the presence and location of emotion-informative facial features, based on differences in low-level visual features (as our first fixation data in the unrestricted, blindspot, and neutral blindspot conditions indicate), and to more global aspects of the face stimulus. Differences in eye movements and thus fixation patterns across emotion categories could also or instead be driven in a top–down fashion related to the observer's knowledge of facial structure (where the different features are in relation to each other) and their current goal—that is, to decide whether the current face is expressing one emotion rather than one or more other, different emotions. The bottom–up drivers of gaze behavior are not limited to extrafoveally presented aspects of the stimulus; there is evidence that foveal processing can also affect where the eyes move to next (Wolf, Belopolsky, & Lappe, 2022), although we do not yet have a complete picture of exactly what features or other aspects of current foveal input can affect subsequent fixation location. In the present study, the high similarities in the fixation patterns between the unrestricted condition and the blindspot and neutral blindspot conditions indicates that foveal processing contributed little if anything to the selection of saccade targets (fixation locations), which leaves extrafoveal stimulus features and top–down knowledge as possible drivers. Differences across expression types in relatively low-level, extrafoveally presented visual features are likely to have played some role in determining where our observers fixated in these three experimental conditions, certainly for first fixations (as evidenced in our data), but perhaps also for subsequent fixations. Nonetheless, the finding that fixations were biased to the same, emotion-informative facial features in the spotlight condition (in which extrafoveal information is absent) as in the other three conditions (in which extrafoveal information about the expression is present) hints at some contribution of top–down knowledge to the selection of saccade targets in those other three conditions, as well.

The gaze decoding analyses and the associated FDMs and marginal FDMs were thus very informative of the spatiotemporal distributions of fixations during facial emotion judgments, but the recurrent quantification analyses additionally revealed information about the temporal *dynamics* of those fixations. Key findings revealed by the RQA were as follows. There were some, though limited, emotion-specific effects on fixation dynamics. Averaged across stimulus presentation conditions, observers refixated parts of fearful and surprised faces slightly but significantly less often than they refixated parts of disgusted, angry, and sad faces (about 42% vs. 44%–46% of the time), and specific sequences of fixations for fearful and surprised faces were less often repeated than for disgusted, angry, and sad faces. In addition, for disgusted faces in the spotlight condition only, observers returned to the same location later in time than they did for the other expression types. The marginal FDMs (Figure 7) and refixation heatmaps (Supplementary Figure S2) indicate that that location of refixation for disgusted face is primarily on and around the philtrum and lower nose. Averaged across all five emotions, observers refixated facial locations in the spotlight condition (in which extrafoveal information was absent) considerably less often than in the other conditions (34% vs. 45%–49% of the time). This was the case whether we considered all trials or correct trials only, although for correct trials with disgusted faces the difference in the number of refixations between the spotlight and neutral blindspot conditions was small and not statistically significant (see Supplementary Materials). The CORM values from the RQA indicated that, when participants’ fixations returned to the same location, they tended to do so sooner in the spotlight condition than in the other three stimulus presentation conditions. Moreover, the determinism values showed that repeating sequences of fixations were more likely for the spotlight condition than for the other three stimulus presentation conditions. Again, these findings were obtained for all trials and for correct trials only.

Our RQA findings showing that differences between the spotlight condition and the other three stimulus presentation conditions are similar to the findings of an experiment reported by Anderson et al. (2013), in which observers viewed indoor and outdoor static scenes. In that experiment, as in our own, recurrence and CORM values were lower and determinism values higher for the gaze-contingent window condition than for natural (unrestricted) viewing, even though their experiment used a substantially larger (5° × 5° square) gaze-contingent window. These results highlight the importance of extrafoveal information for guiding gaze, particularly for reinspecting previously fixated scene locations.

### Limitations

The region of the face covered by the gaze-contingent foveal mask (in the blindspot and neutral blindspot conditions) or window (in the spotlight condition) was small, encircling an area approximately equivalent to the width of the visible eyeball. A circle of 2° diameter was chosen to ensure masking or windowing of the whole foveal region with very little extension into parafovea, as has also been implemented in the foveal-only conditions of two previous studies using gaze-contingent windowing of faces (Caldara et al., 2010; Urtado et al., 2024; although in Caldara et al., it was a Gaussian window with complete opacity at its border, whereas, in Urtado et al., as in the present study, the window was a circle with uniform transparency). But, as the face images were presented at a fixed size, equivalent to viewing a real face at the shorter end (∼45–50 cm) of what Hall (1966) designated the “close phase of personal distance” (∼76–45 cm), we do not know how different our results would be if the images subtended different (especially smaller) visual angles, and thus different (especially larger) regions of the face were masked or windowed. Subsequent research might therefore investigate the impact on emotion recognition and gaze behavior of foveal masking and windowing of faces subtending smaller visual angles.

Another methodological limitation of our study is that the neutral blindspot condition introduced a distortion to the viewed image around the border of the gaze-contingent mask when observers fixated certain parts of the face, especially those parts that differed substantially in their appearance between the emotional and neutral expressions (e.g., the open mouth of a surprised face partially obscured by the corresponding part of the closed neutral expression mouth). We expect this is unlikely to have impacted our results, although we cannot be certain.

A stimulus presentation condition that we could have included but did not is the mirror image of the neutral blindspot condition—that is, a gaze-contingent circular aperture with the facial expression inside and the rest of the image the corresponding emotionally neutral face. We had considered using this additional condition but decided against including it for practical reasons (principally participant fatigue). Yet, this emotional “spotlight” condition has the potential to reveal more about the distinct contributions of foveal and extrafoveal processing of features within faces to emotion recognition and gaze behavior. Thus, future research could implement both the emotional “spotlight” and neutral blindspot conditions in the same experiment.

We did not use dynamic stimuli, despite their greater ecological validity. It is unclear whether our results would have been different if we had used dynamic rather than static facial expressions. Blais et al. (2017) reported that observers fixated on the mouth and (left) eye areas less often and on the nose/face center more often during the recognition of dynamic than static expressions. Yet, Paparelli et al. (2024) found no such differences, which they suggest might be due to two methodological differences between their study and that of Blais et al. (2017). First, Paparelli et al. (2024) used larger face images than did Blais et al. (2017) (14° vertical vs. 5.72° vertical), with larger images affording and possibly requiring greater exploration for accurate emotion classification. Second, the stimuli were presented for 500 ms in the Blais et al. (2017) study but for 1 second in the Paparelli et al. (2024) study, and the shorter presentation time might favor a central fixation strategy for the efficient gathering of task-relevant information. Note that our stimuli subtended visual angles more comparable to that in the study by Paparelli et al. (2024) (23° vertical for the image, 14°–18° vertical for the face itself) and were presented for 3 seconds, thus affording greater opportunity for visual exploration. The small images and relatively brief presentation times in the Blais et al. (2017) study might also explain why they did not find, as we did, that different expressions evoked different gaze patterns.

We also acknowledge some uncertainty over how well our findings will generalize to people of different genders, ages, socioeconomic groups, intelligence, and cultures. Our participants were all young, university-educated adults, predominantly female (80%) and European (86%). Yet, differences in facial emotion recognition have been widely reported in older versus younger adults (for meta-analysis and reviews, see Hayes et al., 2020; Ruffman, Henry, Livingstone, & Phillips, 2008), between females and males (for reviews, see Hall, 1978; Kret & De Gelder, 2012; for a meta-analysis, see Thompson & Voyer, 2014) and across cultures (for a meta-analysis, see Elfenbein & Ambady, 2002; for reviews, see Mesquita & Frijda, 1992; Russell, 1994), and there is evidence of a strong positive association between supramodal emotion recognition ability and general intelligence (Connolly, Lefevre, Young, & Lewis, 2020; Connolly, Young, & Lewis, 2019; Lewis, Lefevre, & Young, 2016). Cross-cultural differences in eye-gaze behavior to faces have also been reported (Blais et al., 2008; Caldara et al., 2010; Fu et al., 2012; Jack et al., 2009; Kelly et al., 2011; Miellet et al., 2013; Senju et al., 2013), as have spatially and temporally distinct face scanning strategies of people from a single (European) culture during emotion classification tasks (Paparelli et al., 2024; Yitzhak et al., 2022). Although our sample consisted of female and male participants of European and East Asian ethnicities, the numbers and proportions of genders and ethnicities were too small to afford meaningful comparisons across these groupings.

## Conclusions

A key overarching finding from this study is that fixation patterns have strong predictive utility even for faces, which are very homogeneous stimuli that engender gaze behavior that is often considered archetypal and routine. More specifically, we provided evidence that, when deciphering emotional expressions on faces, observers’ spatial attention was biased by the viewed emotion such that the emotion category of individual face images was predicted from evoked gaze patterns. Although fixations clustered on and around the eyes, nose, and upper mouth, clear emotion-specific biases in fixation densities (locations weighted by fixation duration) were nevertheless evident, picking out task-informative facial regions. What information was projected to the fovea made little discernable difference to these gaze patterns (or to emotion classification performance). Even first fixations on the face were biased to emotion-specific, task-informative facial regions, suggesting a role for bottom–up, stimulus-driven guidance of eye movements, at least in the initial stages of face processing. Yet, even in the absence of such bottom–up and thus extrafoveally presented drivers of eye movements (i.e., when provided only foveal input from the face), observers’ fixations were biased to the same emotion-specific, task-informative facial regions, suggesting that they know, or came to know over the course of the experiment, what and thus where those diagnostic features are.

## Supplementary Material

Supplement 1

## Footnotes

1 This calculation is based on an average face height of 18 cm (Fang, Clapham, & Chung, 2011) and the fovea subtending 2° of visual angle. Note that definitions of the fovea and its angular size in humans vary in the literature, typically specified as 1°, 1.5°, 1.7°, or 2°, but sometimes even up to 5.2° (e.g., Tuten & Harmening, 2021; Wandell, 1995), depending at least in part on the anatomical features used to define its boundary. For example, the retinal region that is free of rod cells and blood vessels, which is sometimes referred to as the foveola or foveal avascular zone but which others simply refer to as the fovea (e.g., Tuten & Harmening, 2021), corresponds to the central 1° to 1.5° of the visual field, whereas the area of pit in the center of the macula, which is sometimes referred to as the anatomical fovea, corresponds to the central 5° to 5.2° of the visual field. In this study, we were interested in functional differences for facial emotion recognition between foveal and extrafoveal vision, and particularly in differences in the ability to resolve mid- to high spatial frequencies that have been identified as important in facial emotion recognition (e.g., Cassidy, Wiley, Sim, & Hugenberg, 2021; Kumar & Srinivasan, 2011; Smith et al., 2005; Smith & Schyns, 2009; Wylie, Tracy, & Young, 2022). Visual acuity and contrast sensitivity drop off steeply but smoothly from the fovea into the periphery rather than with sudden transitions (Loschky, George, Jian, & Miller, 2005; Robson & Graham, 1981; Rosenholtz, 2016; Wandell, 1995). Thus, following Strasburger et al. (2011), we refer to foveal vision as that below 2° eccentricity, which is also the typical angular size of gaze-contingent masks or windows used in studies examining foveal and or extrafoveal processing of faces or scenes (e.g., Caldara et al., 2010; Miellet et al., 2010; Urtado et al., 2024).

2 Visual crowding is the catastrophic impairment in recognition of objects or detail by clutter, that is, by nearby objects, patterns, or contours (Levi, 2011).

3 Female face IDs: 1, 2, 4, 12, 14, 16. Male face IDs: 3, 7, 9, 20, 21, 24.

4 We did not include expressions of happiness for several related reasons: happiness is the only clearly positive basic emotion, is commonly recognized at very high levels of accuracy and speed when shown in the face to the extent that ceiling effects are often observed, and is thus rarely confused with the other basic emotions (e.g., Calder, Young, et al., 2000; Calvo & Lundqvist, 2008; Kirita & Endo, 1995; Palermo & Coltheart, 2004; Suzuki et al., 2006). There was also a balance to be struck between the number of distinct emotions used and the number of different exemplars of each emotion, to minimise participant fatigue.

5 The “unbiased hit rate” (H_u_) accounts for response biases in classification experiments with multiple response options (Wagner, 1993). H_u_ for each participant is calculated as the squared frequency of correct responses for a target emotion in a particular condition divided by the product of the number of stimuli in that condition representing this emotion and the overall frequency that that emotion category is chosen for that condition. H_u_ ranges from 0 to 1, with 1 indicating that all stimuli representing the target emotion have been correctly identified and that that emotion label has never been falsely selected for a different emotion.
