# Supplementary material for: Distinct contributions of foveal and extrafoveal visual information to emotion judgments and gaze behavior for faces
Source: J Vis. 2025 Jul 2;25(8):4. doi: 10.1167/jov.25.8.4 (PMC12227034; doi:10.1167/jov.25.8.4)
Supplement: Supplement 1 [file jovi-25-8-4_s001.pdf]

Distinct Contributions of Foveal vs. Extrafoveal Visual Information to Emotion Judgements and Gaze Behaviour for Faces

Anthony P. Atkinson, Nazire Duran, Abigail Skraga, Anita Winterbottom, Jack D. Wright

Supplementary Materials

Emotion Classification Performance: Accuracy

Emotion classification accuracy, expressed as hit rates (proportion correct values), are summarized in Supplementary Figure 1. A repeated-measures ANOVA on the proportion correct values revealed significant main effects of stimulus presentation condition, $F(2.42, 82.4) = 59.31$, $p < .001$, $\eta_p^2 = .64$, and emotion, $F(4, 136) = 22.45$, $p < .001$, $\eta_p^2 = .4$, and a significant interaction, $F(7.64, 259.85) = 2.3$, $p = .023$, $\eta_p^2 = .06$. Simple main effects analyses revealed significant effects of stimulus presentation condition for all 5 emotions (all *F*s ≥ 10.66, all *p*s < .001). Paired samples t-tests revealed, for all 5 emotions, reliably lower emotion classification accuracy for the spotlight condition compared to the other 3 stimulus presentation conditions (all *t*s ≥ 8.86, all *p*s < .001, minimum Bonferroni-Holm adjusted α = .0083 for 6 comparisons). There were no other reliable differences in emotion classification accuracy across the stimulus presentation conditions for any of the 5 emotions (all |*t*s| ≤ 0.74, all *p*s ≥ .46). The misclassification data are presented as confusion matrices in Supplementary Table 1.

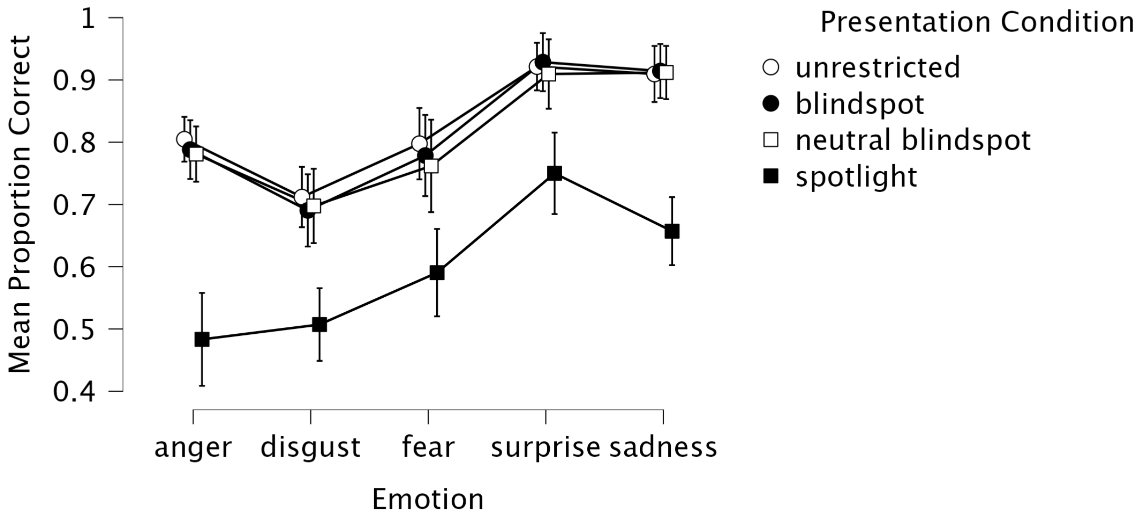

Supplementary Figure 1. Emotion classification accuracy (proportion correct hit rates) as a function of emotion category and stimulus presentation condition. Circles and squares indicate mean values across participants; error bars indicate the 95% confidence intervals.

Stimulus	Response				
	Anger	Disgust	Fear	Surprise	Sadness
	Unrestricted				
Anger	**0.8048**	0.0452	0.0167	0.0024	0.1310
Disgust	0.2619	**0.7119**	0.0024	0.0024	0.0214
Fear	0.0048	0.0381	**0.7976**	0.1452	0.0143
Surprise	0.0024	0.0286	0.0452	**0.9214**	0.0024
Sadness	0.0190	0.0357	0.0286	0.0071	**0.9095**
	Blindspot				
Anger	**0.7881**	0.0714	0.0405	0.0000	0.1000
Disgust	0.3000	**0.6905**	0.0000	0.0024	0.0071
Fear	0.0071	0.0452	**0.7786**	0.1619	0.0071
Surprise	0.0000	0.0286	0.0429	**0.9286**	0.0000
Sadness	0.0143	0.0333	0.0357	0.0024	**0.9143**
	Neutral Blindspot				
Anger	**0.7810**	0.0714	0.0262	0.0024	0.1190
Disgust	0.2714	**0.6976**	0.0024	0.0095	0.0190
Fear	0.0119	0.0690	**0.7619**	0.1214	0.0357
Surprise	0.0024	0.0429	0.0333	**0.9095**	0.0119
Sadness	0.0190	0.0357	0.0286	0.0048	**0.9119**
	Spotlight				
Anger	**0.4833**	0.1024	0.0881	0.0429	0.2833
Disgust	0.3833	**0.5071**	0.0333	0.0357	0.0405
Fear	0.0190	0.1405	**0.5905**	0.2095	0.0405
Surprise	0.0071	0.0405	0.1619	**0.7500**	0.0405
Sadness	0.1405	0.0762	0.0810	0.0452	**0.6571**

Supplementary Table 1. Confusion matrices for emotion classification in each of the 4 stimulus presentation conditions. Data are mean proportion classification rates. Values in bold are for correct responses.

Summary of the confusion matrices

For the unrestricted stimulus presentation condition, disgust was often misclassified as anger, about 26% of the time, but rarely was anger misclassified as disgust (about 4.5% of the time). Fear was misclassified as surprise on around 14.5% of trials, but rarely was surprise misclassified as fear (about 4.5% of the time). Anger was misclassified as sadness on about 13% of trials, but rarely was sadness misclassified as anger (about 1.9% of trials). Sadness was a little more frequently misclassified as disgust (about 3.6% of the time). These patterns of confusion between anger and disgust and between fear and surprise were also evident in the blindspot and neutral 'blindspot' conditions, slightly more so in the blindspot and slightly less so in the neutral 'blindspot' conditions. Accuracy was considerably lower, and the rates of misclassifications therefore considerably higher, in the spotlight condition compared to the other 3 stimulus presentation conditions. Here, misclassifications of disgust as anger increased, to about 38% of trials, as did the misclassifications of anger as disgust, to about 10% of trials, and the misclassification of anger as sadness increased even more so, to about 28% of trials. Misclassifications of fear as surprise increased, to about 21% of trials, with an even bigger increase in misclassifications of surprise as fear, to about 16% of trials. There were also notable increases in misclassifications of fear as disgust (to about 14% of trials), sadness as anger (to about 14% of trials), or disgust (about 7.6% of trials), or fear (about 8% of trials).

Additional FDM Correlation Analyses

In the main paper we report correlation analyses for the fixation density maps (FDMs) generated from the fixation data for the unrestricted stimulus presentation condition, comparing within-emotion correlations with between-emotion correlations. Here we report the same correlation analyses for the other stimulus presentation conditions, which show the same pattern of results as those for the unrestricted condition.

The FDMs associated with the same emotions when calculated based on all fixations and on first fixations were more like each other than were the FDMs associated with different emotions when calculated based on all fixations and on a first fixations. This was true for all stimulus presentation conditions. When based on fixations from all trials, within-emotion correlations between all-fixation FDMs and first-fixation FDMs (panels A and B in Figure 5-7) were on average significantly larger than between-emotion correlations between

all-fixation FDMs and first-fixation FDMs; blindspot: $t(34) = 7.74$, $p < .001$ (within-emotion $M = .73$, $SD = .088$, between-emotion $M = .683$, $SD = .085$); neutral ‘blindspot’: $t(34) = 9.55$, $p < .001$ (within-emotion $M = .715$, $SD = .102$, between-emotion $M = .659$, $SD = .104$); spotlight: $t(34) = 7.42$, $p < .001$ (within-emotion $M = .441$, $SD = .124$, between-emotion $M = .389$, $SD = .105$) Likewise, when based on trials with correct responses only, within-emotion correlations between all-fixation FDMs and first-fixation FDMs (panels E and F in Figures 5-7) were on average significantly larger than between-emotion correlations between all-fixation FDMs and first-fixation FDMs; blindspot: $t(34) = 7.74$, $p < .001$ (within-emotion $M = .706$, $SD = .087$, between-emotion $M = .648$, $SD = .08$); neutral ‘blindspot’: $t(34) = 9.72$, $p < .001$ (within-emotion $M = .683$, $SD = .137$, between-emotion $M = .625$, $SD = .127$); spotlight: $t(34) = 9.02$, $p < .001$ (within-emotion $M = .391$, $SD = .108$, between-emotion $M = .313$, $SD = .098$).

Correlation analyses also confirmed that the FDMs associated with the same emotions when calculated based on fixations from all trials and fixations from correct-only trials were more like each other than were the FDMs associated with different emotions when calculated based on fixations from all trials and fixations from correct-only trials. Again, this was true for all stimulus presentation conditions. When based on all fixations, within-emotion correlations between all-trial FDMs and correct-only trial FDMs (panels A and E in Figures 5-7) were on average significantly larger than between-emotion correlations between all-trial FDMs and correct-only trial FDMs; blindspot: $t(34) = 20.41$, $p < .001$ (within-emotion $M = .985$, $SD = .012$, between-emotion $M = .876$, $SD = .046$); neutral ‘blindspot’: $t(34) = 19.32$, $p < .001$ (within-emotion $M = .977$, $SD = .059$, between-emotion $M = .868$, $SD = .069$); spotlight: $t(34) = 15.47$, $p < .001$ (within-emotion $M = .92$, $SD = .059$, between-emotion $M = .763$, $SD = .091$). Likewise, when based on first fixations only, within-emotion correlations between all-trial FDMs and correct-only trial FDMs (panels B and F in Figures 5-7) were on average significantly larger than between-emotion correlations between all-trial FDMs and correct-only trial FDMs; blindspot: $t(34) = 14.55$, $p < .001$ (within-emotion $M = .965$, $SD = .029$, between-emotion $M = .755$, $SD = .106$); neutral ‘blindspot’: $t(34) = 15.92$, $p < .001$ (within-emotion $M = .956$, $SD = .071$, between-emotion $M = .728$, $SD = .114$); spotlight: $t(34) = 16.56$, $p < .001$ (within-emotion $M = .883$, $SD = .08$, between-emotion $M = .619$, $SD = .136$).

All fixations, all trials		
	t	*p*
(unrestricted vs. blindspot) > (unrestricted vs. neutral ‘blindspot’)	0.63	.53
(unrestricted vs. blindspot) > (unrestricted vs. spotlight)	14.93	< .001
(unrestricted vs. blindspot) > (blindspot vs. neutral ‘blindspot’)	-0.62	.54
(unrestricted vs. blindspot) > (blindspot vs. spotlight)	15.09	< .001
(unrestricted vs. blindspot) > (neutral ‘blindspot’ vs. spotlight)	12.33	< .001
(unrestricted vs. neutral ‘blindspot’) > (unrestricted vs. spotlight)	13.15	< .001
(unrestricted vs. neutral ‘blindspot’) > (blindspot vs. neutral ‘blindspot’)	-1.14	.26
(unrestricted vs. neutral ‘blindspot’) > (blindspot vs. spotlight)	13.05	< .001
(unrestricted vs. neutral ‘blindspot’) > (neutral ‘blindspot’ vs. spotlight)	10.72	< .001
(unrestricted vs. spotlight) > (blindspot vs. neutral ‘blindspot’)	-13.27	< .001
(unrestricted vs. spotlight) > (blindspot vs. spotlight)	-1.19	.24
(unrestricted vs. spotlight) > (neutral ‘blindspot’ vs. spotlight)	-4.09	< .001
(blindspot vs. neutral ‘blindspot’) > (blindspot vs. spotlight)	13.57	< .001
(blindspot vs. neutral ‘blindspot’) > (neutral ‘blindspot’ vs. spotlight)	10.82	< .001
(blindspot vs. spotlight) > (neutral ‘blindspot’ vs. spotlight)	-2.93	.006

All fixations, correct trials		
	t	*p*
(unrestricted vs. blindspot) > (unrestricted vs. neutral ‘blindspot’)	0.79	.43
(unrestricted vs. blindspot) > (unrestricted vs. spotlight)	16.03	< .001
(unrestricted vs. blindspot) > (blindspot vs. neutral ‘blindspot’)	-0.19	.85
(unrestricted vs. blindspot) > (blindspot vs. spotlight)	15.71	< .001
(unrestricted vs. blindspot) > (neutral ‘blindspot’ vs. spotlight)	12.97	< .001
(unrestricted vs. neutral ‘blindspot’) > (unrestricted vs. spotlight)	12.95	< .001
(unrestricted vs. neutral ‘blindspot’) > (blindspot vs. neutral ‘blindspot’)	-0.96	.34
(unrestricted vs. neutral ‘blindspot’) > (blindspot vs. spotlight)	12.82	< .001
(unrestricted vs. neutral ‘blindspot’) > (neutral ‘blindspot’ vs. spotlight)	11.28	< .001
(unrestricted vs. spotlight) > (blindspot vs. neutral ‘blindspot’)	-13.6	< .001
(unrestricted vs. spotlight) > (blindspot vs. spotlight)	-1.35	.19
(unrestricted vs. spotlight) > (neutral ‘blindspot’ vs. spotlight)	-3.25	.003
(blindspot vs. neutral ‘blindspot’) > (blindspot vs. spotlight)	13.84	< .001
(blindspot vs. neutral ‘blindspot’) > (neutral ‘blindspot’ vs. spotlight)	11.97	< .001
(blindspot vs. spotlight) > (neutral ‘blindspot’ vs. spotlight)	-2.39	.023

First fixations, all trials		
	t	*p*
(unrestricted vs. blindspot) > (unrestricted vs. neutral ‘blindspot’)	0.03	.98
(unrestricted vs. blindspot) > (unrestricted vs. spotlight)	7.8	< .001
(unrestricted vs. blindspot) > (blindspot vs. neutral ‘blindspot’)	-1.21	.24
(unrestricted vs. blindspot) > (blindspot vs. spotlight)	6.51	< .001
(unrestricted vs. blindspot) > (neutral ‘blindspot’ vs. spotlight)	6.43	< .001
(unrestricted vs. neutral ‘blindspot’) > (unrestricted vs. spotlight)	8.77	< .001
(unrestricted vs. neutral ‘blindspot’) > (blindspot vs. neutral ‘blindspot’)	-1.41	.17
(unrestricted vs. neutral ‘blindspot’) > (blindspot vs. spotlight)	7.08	< .001
(unrestricted vs. neutral ‘blindspot’) > (neutral ‘blindspot’ vs. spotlight)	6.92	< .001
(unrestricted vs. spotlight) > (blindspot vs. neutral ‘blindspot’)	-8.36	< .001
(unrestricted vs. spotlight) > (blindspot vs. spotlight)	-1.74	.09
(unrestricted vs. spotlight) > (neutral ‘blindspot’ vs. spotlight)	-2.93	.006
(blindspot vs. neutral ‘blindspot’) > (blindspot vs. spotlight)	7.12	< .001
(blindspot vs. neutral ‘blindspot’) > (neutral ‘blindspot’ vs. spotlight)	7.09	< .001
(blindspot vs. spotlight) > (neutral ‘blindspot’ vs. spotlight)	-0.85	.4

First fixations, correct trials		
	t	*p*
(unrestricted vs. blindspot) > (unrestricted vs. neutral ‘blindspot’)	-0.14	.89
(unrestricted vs. blindspot) > (unrestricted vs. spotlight)	8.43	< .001
(unrestricted vs. blindspot) > (blindspot vs. neutral ‘blindspot’)	-1.09	.28
(unrestricted vs. blindspot) > (blindspot vs. spotlight)	7.43	< .001
(unrestricted vs. blindspot) > (neutral ‘blindspot’ vs. spotlight)	7.02	< .001
(unrestricted vs. neutral ‘blindspot’) > (unrestricted vs. spotlight)	8.57	< .001
(unrestricted vs. neutral ‘blindspot’) > (blindspot vs. neutral ‘blindspot’)	-1.15	.26
(unrestricted vs. neutral ‘blindspot’) > (blindspot vs. spotlight)	7.41	< .001
(unrestricted vs. neutral ‘blindspot’) > (neutral ‘blindspot’ vs. spotlight)	7.23	< .001
(unrestricted vs. spotlight) > (blindspot vs. neutral ‘blindspot’)	-8.2	< .001
(unrestricted vs. spotlight) > (blindspot vs. spotlight)	-0.99	.33
(unrestricted vs. spotlight) > (neutral ‘blindspot’ vs. spotlight)	-2.01	.05
(blindspot vs. neutral ‘blindspot’) > (blindspot vs. spotlight)	7.48	< .001
(blindspot vs. neutral ‘blindspot’) > (neutral ‘blindspot’ vs. spotlight)	7.29	< .001
(blindspot vs. spotlight) > (neutral ‘blindspot’ vs. spotlight)	-0.85	.4

Supplementary Table 2. Results of paired-samples t-tests (*df* = 34) comparing within-emotion FDM correlations across all pairwise combinations of the 4 stimulus presentation conditions, separately for each category of fixations.

Recurrence Quantification Analyses (RQA) Only for Trials with Correct Responses

Note that these data and associated analyses are for 33 of the 35 participants only; the remaining 2 participants had missing data in one condition (i.e., no trials with a correct response in that condition).

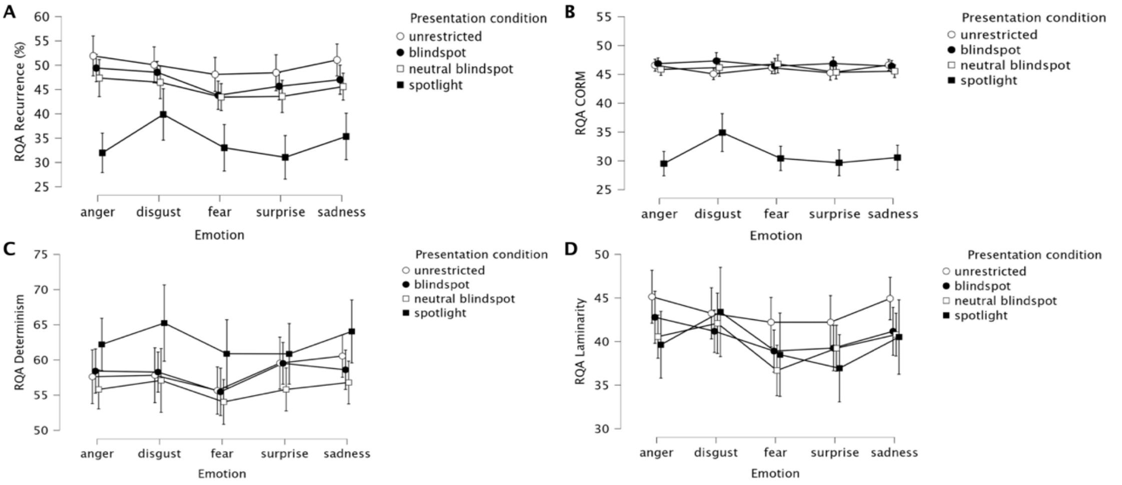

Supplementary Figure 2. RQA measures of recurrence (panel A), CORM (panel B), determinism (panel C), and laminarity (panel D), as a function of emotion and stimulus presentation condition, only for trials with correct responses. Circles and squares indicate mean values across participants; error bars indicate the 95% confidence intervals.

Recurrence

The recurrence measures are summarized in Supplementary Figure 1A. The repeated-measures ANOVA revealed significant main effects of stimulus presentation condition, $F(1.89, 60.52) = 16.46$, $p < .001$, $\eta_p^2 = .34$, and emotion, $F(4, 128) = 9.4$, $p < .001$, $\eta_p^2 = .23$. These main effects were modified by an interaction between stimulus condition and emotion, $F(12, 384) = 2.93$, $p < .001$, $\eta_p^2 = .08$.

Pairwise comparisons for the factor stimulus condition revealed the following. There were reliably lower recurrence values for the spotlight condition (*M* = 34.26, *SD* = 10.85)

compared to the other three conditions: the blindspot condition (*M* = 46.9, *SD* = 11.48), *t*(32) = 5.34, $p < .001$, *d* = 0.93, 95% CI [0.52, 1.33], the unrestricted condition (*M* = 49.93, *SD* = 13.23), *t*(32) = 4.92, $p < .001$, *d* = 0.86, 95% CI [0.45, 1.25], and the neutral 'blindspot' condition (*M* = 45.3, *SD* = 13.24), *t*(32) = 3.67, $p < .001$, *d* = 0.64, 95% CI [0.26, 1.01]. In other words, on trials in which participants made correct emotion classification responses, they refixated facial locations in the spotlight condition (in which extrafoveal information was absent) about 34% of the time, on average, which is considerably less often than the 45-50% refixations, on average, for the other conditions (in which extrafoveal information was present). The recurrence values for the neutral 'blindspot' condition were also reliably lower than those for the unrestricted condition, *z*(32) = 3.14, *p* = .001, r_{rb} = 0.63, 95% CI [0.33, 0.81] The remaining two pairwise comparisons were not significant (both *t*s ≤ 1.57, uncorrected *p*s ≥ .13).

Pairwise comparisons for the factor emotion revealed reliably higher recurrence values for disgusted faces (*M* = 46.25, *SD* = 9.36) compared to fearful faces (*M* = 42.1, *SD* = 9.07), *t*(32) = 5.41, $p < .001$, *d* = 0.94, 95% CI [0.52, 1.35], and surprised faces (*M* = 42.19, *SD* = 9.53), *t*(32) = 4.25, $p < .001$, *d* = 0.74, 95% CI [0.35, 1.12]. There were also higher recurrence values for angry faces (*M* = 45.17, *SD* = 9.69) compared to fearful faces, *t*(32) = 3.21, *p* = .003, *d* = 0.56, 95% CI [0.19, 0.92], and surprised faces, *t*(32) = 3.07, *p* = .004, *d* = 0.53, 95% CI [0.17, 0.9]. And there were higher recurrence values for sad faces compared to surprised faces, *t*(32) = 3.58, *p* = .001, *d* = 0.62, 95% CI [0.25, 0.99], and fearful faces, *t*(32) = 3.49, *p* = .001, *d* = 0.61, 95% CI [0.23, 0.98]. The remaining 3 pairwise comparisons were not significant (|*t*s| ≤ 2.05, uncorrected *p*s ≥ .05). In other words, participants refixated parts of fearful and surprised faces less often (about 42% of the time, on average) than they refixated parts of disgusted (~46%), angry (~45%), and sad (~45%) faces.

Simple main effect analyses to follow up the interaction revealed significant main effects of presentation condition for all 5 emotions (anger: *F*(3) = 21.75, $p < .001$; disgust: *F*(3) = 4.99, *p* = .003; fear: *F*(3) = 11.6, $p < .001$; surprise: *F*(3) = 16.56, $p < .001$; sadness: *F*(3) = 12.53, $p < .001$). Pairwise comparisons across stimulus presentation conditions for each emotion separately, Bonferroni-Holm corrected for 6 comparisons, showed that for disgusted faces, recurrence values were lower in the spotlight condition than in the blindspot condition, *t*(32) = 3.08, *p* = .004, *d* = 0.54, 95% CI [0.17, 0.9], and in the unrestricted condition, *t*(32) = 2.71, *p* = .011, *d* = 0.47, 95% CI [0.11, 0.83]. The latter comparison was not

statistically significant following correction for multiple comparisons (relevant Bonferroni-Holm adjusted $\alpha = .01$), but the effect size was greater than the minimum detectable effect size of $d_z = 0.429$ produced by our sensitivity analysis (see Method). For the other 4 emotions, by contrast, recurrence values were lower in the spotlight condition than in all 3 other stimulus presentation conditions (all *t*s > 3.2, *p*s < .004), which themselves did not differ.

Centre of Recurrent Mass (CORM)

The CORM measures are summarized in Supplementary Figure 1B. The repeated-measures ANOVA revealed a significant main effect of stimulus presentation condition, $F(1.32, 42.25) = 171.39$, $p < .001$, $\eta_p^2 = .84$, and a significant interaction, $F(6.54, 209.42) = 4.18$, $p < .001$, $\eta_p^2 = .12$. The main effect of emotion was not significant, $F(2.75, 87.85) = 2.56$, $p = .065$, $\eta_p^2 = .07$. There were reliably and substantially smaller CORM values for the spotlight condition ($M = 31.02$, $SD = 4.53$) compared to the other three conditions: the unrestricted condition ($M = 45.94$, $SD = 3.2$), $t(32) = 14.4$, $p < .001$, $d = 2.51$, 95% CI [1.8, 3.2], the blindspot condition ($M = 46.79$, $SD = 3.31$), $t(32) = 14.31$, $p < .001$, $d = 2.49$, 95% CI [1.79, 3.18], and the neutral 'blindspot' condition ($M = 45.96$, $SD = 3.41$), $t(32) = 12.92$, $p < .001$, $d = 2.25$, 95% CI [1.6, 2.89]. (All other $|ts| \leq 2.17$, uncorrected *p*s ≥ .038.) In other words, when participants' fixations returned to the same location, they tended to do so sooner in the spotlight condition than in the control, blindspot or neutral 'blindspot' conditions.

Simple main effects analyses to follow up the significant interaction revealed significant effects of emotion for the spotlight condition, $F(4) = 5.69$, $p < .001$, but not for the other stimulus presentation conditions (*F*s ≤ 2.35, *p*s ≥ .058). Pairwise comparisons for the spotlight condition revealed reliably larger CORM values for disgusted faces ($M = 34.9$, $SD = 9.11$) compared to all the other 4 emotional expressions: surprised faces ($M = 29.67$, $SD = 5.96$), $z(32) = 3.69$, $p < .001$, $r_{rb} = 0.74$, 95% CI [0.5, 0.87], sad faces ($M = 30.57$, $SD = 5.67$), $z(32) = 3.23$, $p < .001$, $r_{rb} = 0.64$, 95% CI [0.36, 0.82], angry faces ($M = 29.52$, $SD = 5.74$), $z(32) = 3.05$, $p = .002$, $r_{rb} = 0.61$, 95% CI [0.3, 0.8], and fearful faces ($M = 30.43$, $SD = 5.74$), $z(32) = 2.71$, $p = .006$, $r_{rb} = 0.54$, 95% CI [0.21, 0.76]. The remaining pairwise comparisons were not significant (all $|ts| < 1$, uncorrected *p*s ≥ .35). In other words, in the spotlight condition only, participants' refixations of facial regions tended to be further apart in time for disgusted faces than for angry, fearful, surprised or sad faces (for disgusted faces, they returned to the same location later in time than they did for the other expression types).

Determinism

The determinism measures are summarized in Supplementary Figure 1C. The repeated-measures ANOVA revealed significant main effects of stimulus presentation condition, $F(1.98, 63.42) = 4.26$, $p = .019$, $\eta_p^2 = .12$, and emotion, $F(4, 128) = 3.11$, $p = .018$, $\eta_p^2 = .09$. The interaction was not significant, $F(12, 384) = 0.61$, $p = .83$, $\eta_p^2 = .02$. Pairwise comparisons for the factor stimulus presentation condition revealed reliably larger determinism values for the spotlight condition ($M = 62.65$, $SD = 8.3$) compared to the neutral 'blindspot' condition ($M = 55.91$, $SD = 9.03$), $t(32) = 2.83$, $p = .008$, $dz = 0.49$, 95% CI [0.13, 0.85]. Determinism values were also larger for the spotlight condition compared to the blindspot condition ($M = 58.06$, $SD = 7.01$), $t(32) = 2.54$, $p = .016$, $dz = 0.44$, 95% CI [0.08, 0.8], though this comparison did not survive correction for multiple comparisons (relevant Bonferroni-Holm adjusted α = .01). The remaining pairwise comparisons were not significant (all $|ts| \leq 1.7$, ps ≥ .099). Pairwise comparisons for the factor emotion revealed reliably smaller determinism values for fearful faces ($M = 56,51$, $SD = 6.73$) than for sad faces ($M = 60.0$, $SD = 6.46$), $t(32) = 3.69$, $p < .001$, $dz = 0.64$, 95% CI [0.26, 1.01]. Determinism values were also smaller for fearful faces than for disgusted faces ($M = 59.61$, $SD = 7.59$), $t(32) = 2.55$, $p = .0157$, $dz = 0.44$, 95% CI [0.08, 0.8] and surprised faces ($M = 58.94$, $SD = 6.05$), $t(32) = 2.06$, $p = .047$, $dz = 0.36$, 95% CI [0.004, 0.71], though these comparisons did not survive correction for multiple comparisons (relevant Bonferroni-Holm adjusted α = .0056, and α = .0063, respectively). (All other ts ≤ |1.81|, ps ≥ .08.)

Laminarity

The laminarity measures are summarized in Supplementary Figure 1D. The repeated-measures ANOVA revealed a significant main effect of emotion, $F(4, 128) = 6.99$, $p < .001$, $\eta_p^2 = .18$. The main effect of stimulus presentation condition and the interaction were not significant (Fs ≤ 1.52, ps ≥ .18). The main effect of emotion reflected reliably smaller laminarity values for surprised faces ($M = 39.41$, $SD = 8.81$) and fearful faces ($M = 39.07$, $SD = 8.71$) compared to all 3 other emotional expressions: anger ($M = 42.03$, $SD = 8.53$) > surprise, $t(32) = 3.06$, $p = .004$, $dz = 0.53$, 95% CI [0.16, 0.89]; anger > fear, $t(32) = 3.5$, $p = .001$, $dz = 0.61$, 95% CI [0.23, 0.98]; sadness ($M = 41.85$, $SD = 8.69$) > surprise, $t(32) = 3.22$, $p = .003$, $dz = 0.56$, 95% CI [0.19, 0.92]; sadness > fear, $t(32) = 4.19$, $p < .001$, $dz = 0.73$, 95% CI [0.34, 1.11]; disgust ($M = 42.48$, $SD = 8.6$) > surprise, $t(32) = 2.86$, $p = .008$, $dz = 0.5$, 95% CI [0.13, 0.86]; disgust > fear, $t(32) = 3.56$, $p = .001$, $dz = 0.62$, 95% CI [0.24, 0.99]. (All other $|ts| < 1$,

*p*s $\geq$ .48.) In other words, for fearful and surprised faces, specific sequences of fixations were less often repeated than for angry, disgusted, and sad faces.

Refixation Heatmaps

As mentioned in the main paper, we generated refixation heatmaps for each emotional expression category (collapsed over stimulus presentation conditions) to highlight those regions of the face that received the most refixations. As with the RQA, refixations were defined as two or more fixations within a trial that were within 2.5^{o} visual angle of each other. Creation of the refixation heatmaps involved starting with a blank map the same dimensions as the face images, in which each pixel was assigned a value of 0. A value of 1 was then added to the value of each pixel corresponding to the location of fixations within a trial that qualified as refixations. Thus, summed over all trials for a given emotion, regions of the image with larger pixel values indicate areas of the image refixated more often than areas comprised of pixels with lower values. These condition-specific refixation maps were then smoothed with the same two-dimensional Gaussian kernel (σ = 13 pixels) as were the FDMs. We also created marginal refixation heatmaps by subtracting the grand mean of all unsmoothed refixation heatmaps from each unsmoothed emotion-specific refixation heatmap, thus controlling for spatial biases common across all image categories. These marginal refixation heatmaps were then smoothed with the same two-dimensional Gaussian kernel. The resulting (smoothed) refixation heatmaps and marginal heatmaps are shown in Supplementary Figure 2.

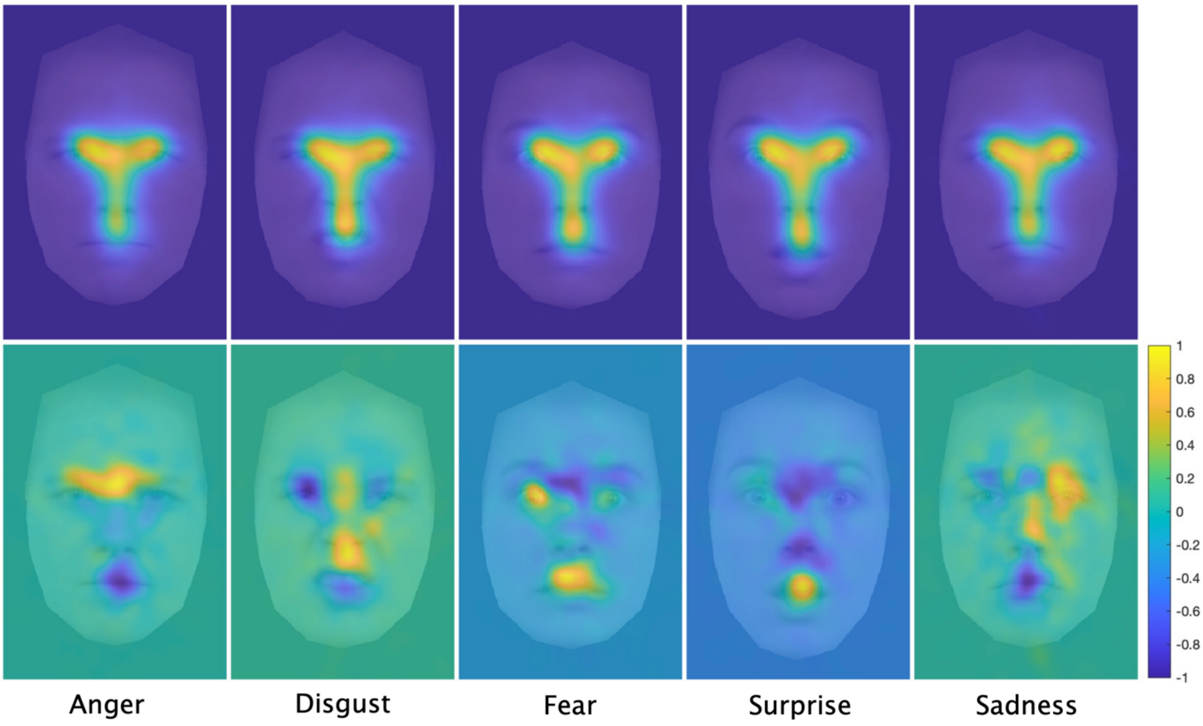

Supplementary Figure 2. Refixation heatmaps (top row) and marginal refixation heatmaps (bottom row), for all trials for each emotion category, collapsed over stimulus presentation condition, overlaid on an average face for the corresponding emotion. For the raw refixation heatmaps (top row), the brighter (yellower) colours indicate regions of the face that received more refixations (defined as two or more fixations within a trial that were within 2.5^{o} visual angle of each other). The scale applies to the marginal refixation heatmaps, where positive values (warmer colour) indicate regions with higher-than-average refixation density, and negative values (cooler colour) represent regions with lower-than-average refixation density.

Analyses with Stimulus Condition Order

We tested the participants in a balanced order across stimulus-presentation conditions to avoid order effects. Yet, as one reviewer pointed out, it is possible that order effects in terms of task performance and or fixation behaviour are indeed evident in certain subgroups of participants. For example, the participants who underwent the unrestricted condition first, thus seeing the full visible facial expression, could have gained some advantage as compared to participants who began with one of the other conditions, especially the spotlight condition. Conversely, the participants who began with the difficult spotlight condition could have been hindered compared to those who began with one of the other conditions. And these two subgroups could have adopted different fixation strategies.

We therefore aimed to test for such order effects. However, the large number of stimulus condition (block) orders used across the 35 participants (22) and thus the very low number of participants per block order (mean = 1.6) means that it is not possible to perform any statistical analyses using all block orders as levels of a block order factor. So, we instead redid some of our analyses using block order divided into smaller numbers of levels.

First, we redid the accuracy, response time, and RQA analyses, this time using data from only those participants who underwent the unrestricted viewing condition first (N = 11) and those participants who underwent the spotlight condition first (N = 8). These were mixed-design repeated measures ANOVAs, with block order as the between-subjects variable (2 levels/groups). For both the accuracy and response time data, the main effect of block order was not significant, and nor were any of the interactions involving block order (all *F*s < 2.35, *p*s > .13). Similarly, for the RQA data, none of the block order main effects for the 4 different parameters, nor any of the interactions involving block order, were significant (all *F*s < 1.3, *p*s > .28).

Second, we redid the accuracy, response time, and RQA analyses, this time using data from only those participants who underwent the unrestricted viewing condition either first or second and the spotlight condition third or fourth (N = 12) and those participants who underwent the spotlight condition first or second and the unrestricted viewing condition either third or fourth (N = 14). Again, these were mixed-design repeated measures ANOVAs, with block order as the between-subjects variable (2 levels/groups). And again, for both the accuracy and response time data, the main effect of block order was not significant, and nor were any of the interactions involving block order (all *F*s < 2.1, *p*s > .05). For the RQA data, there was one significant interaction involving block order. The Emotion x Block Order interaction on the laminarity data, $F(4, 96) = 2.66$, $p = .037$, $\eta_p^2 = .1$, reflected marginally smaller laminarity values for surprised faces for the second group (*M* = 39.13, *SD* = 7.99) compared to the first group (*M* = 42.75, *SD* = 9.41) – but this difference was not statistically significant, $t(24) = 1.06$, $p = .3$, $d = 0.42$ – and more equal laminarity values between groups for the other emotions. None of the other interactions involving block order, and none of the block order main effects, were significant (all *F*s < 3.28, *p*s > .06).

Third, we separated all participants into 2 groups: those who completed the unrestricted viewing condition before completing the spotlight condition (N = 18) and those who completed the spotlight condition before completing the unrestricted viewing condition

(N = 17). Then, we compared the performance of these 2 groups on the spotlight condition only. If the order effects suggested by the reviewer are in play, we would expect participants in the first group to perform better in the spotlight condition than participants in the second group. This was not the case. For accuracy (unbiased hit rates), group 1 participants (*M* = 0.439, *SD* = 0.12) were marginally more accurate than group 2 participants (*M* = 0.373, *SD* = 0.156), but this difference was not statistically significant, $t(33) = 1.41$, $p = .168$, $d = 0.477$. Similarly, for response times, group 1 participants (*M* = 4.38, *SD* = 0.7) were marginally faster than group 2 participants (*M* = 4.57, *SD* = 0.56), but this difference was not statistically significant, $t(33) = 0.88$, $p = .384$, $d = 0.299$. Mixed-design repeated-measures ANOVAs further confirmed that there were no significant accuracy or response-time differences between these two groups for the spotlight condition as a function of the emotion categories (all *F*s < 2.0, *p*s > .16). We also performed group comparison statistical tests on the RQA data, and found that participants in the first group (the unrestricted viewing condition before the spotlight condition) had smaller CORM values on average (*M* = 29.56, *SD* = 3.16) than participants in the second group (the spotlight condition before the unrestricted viewing condition: *M* = 32.38, *SD* = 3.24), $t(33) = 2.24$, $p = .032$, $d = 0.76$. There were no significant differences between these two groups for the other 3 RQA parameters (all *t*s < 1.2, *p*s > .24). In other words, participants' refixations of facial regions tended to be closer together in time for those who received the unrestricted condition *before* the spotlight condition than for those who received the unrestricted condition *after* the spotlight condition.

Finally, we created FDMs and marginal FDMs for the unrestricted and spotlight stimulus presentation conditions, separately for each of the two larger subgroups. These allow a visual assessment of any differences in fixation patterns between participants who received the unrestricted condition *before* the spotlight condition and those who received the unrestricted condition *after* the spotlight condition. The marginal FDMs, calculated from all fixations (correct and incorrect trials together), are shown in Supplementary Figure 3.

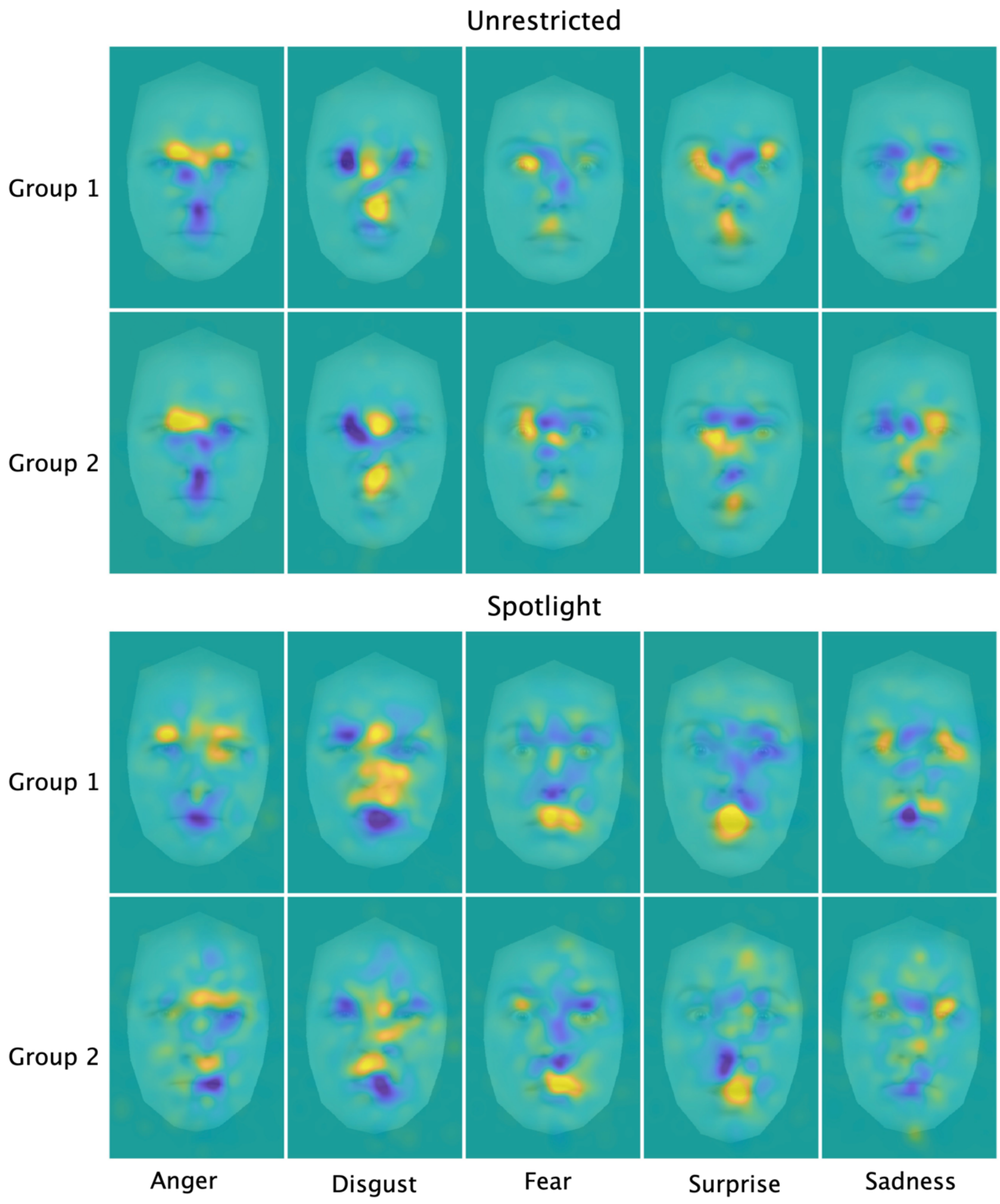

Supplementary Figure 3. Marginal FDMs for the unrestricted (top 2 rows) and spotlight (bottom 2 rows) stimulus presentation conditions, calculated separately for each of 2 subgroups of participants: those who received the unrestricted condition *before* the spotlight viewing condition (group 1) and those who received the unrestricted condition *after* the spotlight condition (group 2). These marginal FDMs are for all fixations, regardless of whether the participant's emotion classification response was correct.

Caution must be exercised in interpreting the results of these new analyses given the small and slightly uneven group sizes and claims around null effects (‘the absence of evidence is not evidence of absence’). Nonetheless, these analyses lead us to conclude that there is no clear evidence that the order in which participants received the full face (unrestricted viewing condition) versus the severely restricted face (spotlight condition) influenced emotion classification task performance. But there is evidence of small differences in patterns of fixation on the faces as a function of the order in which participants received the unrestricted and spotlight conditions. These differences are most evident in the marginal FDMs (Supplementary Figure 3). Yet, the similarities in the fixation patterns between these two groups appear greater than the differences: in particular, the same emotion-specific biases in fixation patterns, which tend to pick out emotion-relevant facial features, are clearly evident.

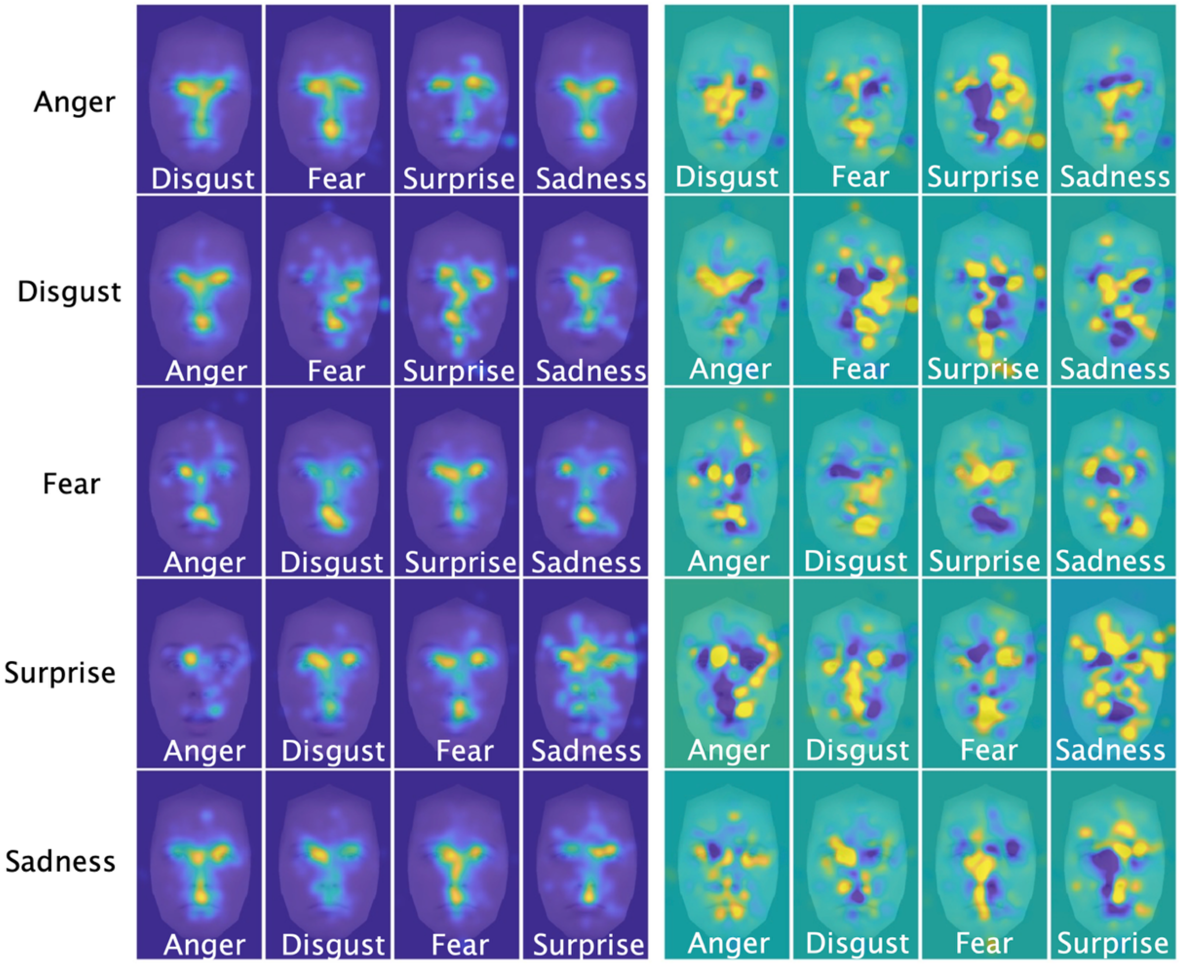

Supplementary Figure 4. FDMs (left-hand panel) and marginal FDMs (right-hand panel) for incorrect-response trials, showing fixation densities (FDMs) and fixation density differences

(relative to the average; marginal FDMs) for each specific misclassification. Each expressed emotion category is represented on a separate row, identified by the black font labels; the misclassifications are indicated by the white font labels overlaid on the individual images.
